# LDOC1 connects histone H2B monoubiquitination to tumor cell plasticity in non-small cell lung cancer

**DOI:** 10.1186/s12964-025-02607-z

**Published:** 2026-01-03

**Authors:** Hsien-Neng Huang, Pin-Feng Hung, Yi-Ta Tsai, En-Ting Liu, Tai-Lung Cha, Ya-Pin Chen, Wen-Tsan Weng, Chiao-Yin Sun, Wei-Hsuan Yu, Hau-Lun Cheng, Chia-Huei Lee

**Affiliations:** 1 Department of Pathology, National Taiwan University Hospital Hsin-Chu Branch, No. 2, Sec. 1, Shengyi Rd, Zhubei City, Hsinchu County 302058 Taiwan; 2 Department and Graduate Institute of Pathology, College of Medicine, National Taiwan University, No. 1, Sec. 1, Jen-Ai Road, Zhongzheng District, Taipei City, 10051 Taiwan; 3 National Institute of Cancer Research, National Health Research Institutes, No. 35, Keyan Rd, Zhunan, Miaoli County 35053 Taiwan; 4 Graduate Institute of Life Sciences, National Defense Medical University, No. 161, Sec. 6, Minquan East Road, Neihu District, Taipei City, 11490 Taiwan; 5 Department of Nephrology, Keelung Chang Gung Memorial Hospital, No. 222, Maijin Rd., Anle Dist, Keelung City, Taiwan; 6 Institute of Biochemistry and Molecular Biology, College of Medicine, National Taiwan University, No. 1, Sec. 1, Jen-Ai Road, Zhongzheng District, Taipei City, 10051 Taiwan

**Keywords:** Non-small cell lung cancer (NSCLC), Leucine zipper downregulated in cancer 1 (LDOC1), H2B monoubiquitination (H2Bub1), Epigenetics, Epithelial–mesenchymal plasticity, Chromatin remodeling, Spread through air spaces (STAS)

## Abstract

**Background:**

Non-small cell lung cancer (NSCLC) remains a leading cause of cancer-related mortality, partly because epigenetic dysregulation drives tumor progression and metastasis. We previously showed that leucine zipper downregulated in cancer 1 (LDOC1) modulates the metastatic potential of NSCLC cells. The structural features of LDOC1 suggest that it can interact with nuclear histones, and although it lacks a canonical nuclear localization signal, it predominantly localizes to the nucleus in NSCLC cells; its loss causes broad transcriptomic changes, supporting a role for LDOC1 as an epigenetic regulator acting through histone modifications.

**Methods:**

The levels of histone proteins were assessed in NSCLC cell lines with either LDOC1 knockdown or ectopic expression. Transcriptomic profiling, ChIP-seq, ATAC-seq, MNase digestion assays, flow cytometry, coimmunoprecipitation, proximity ligation assays, immunofluorescence staining, immunohistochemistry, and functional assays were conducted. Clinical relevance was analyzed with archived NSCLC samples and using datasets from the UCSC Xena database.

**Results:**

LDOC1 interacts with histone H2B and H2Bub1 as well as with PSMA1 to promote their proteasomal degradation, thereby limiting global H2Bub1 levels. Despite increasing global H2Bub1 abundance, LDOC1 knockdown caused a pronounced loss of chromatin-bound H2Bub1 and enhanced chromatin compaction, effects that were partially mediated by the LDOC1–THAP12 interaction. THAP12 overexpression increased LDOC1 recovery in nuclear histone fractions. Integrated transcriptomic and epigenomic analyses revealed that the LDOC1–H2Bub1 axis regulates key metastasis-related genes involved in cytoskeletal remodeling, cell adhesion, and epithelial–mesenchymal (E–M) transition. Functionally, LDOC1 loss enhanced TGF-β–induced E–M plasticity, promoted a hybrid E/M phenotype, reduced adhesion, and altered migration dynamics. In clinical samples, H2Bub1 was significantly upregulated in spread through air spaces (STAS) and inversely correlated with LDOC1 expression. High H2Bub1 expression predicted shorter progression-free survival in EGFRᵂᵀ NSCLC patients receiving chemotherapy, and TCGA data linked LDOC1 downregulation to KRAS-mutant lung adenocarcinoma.

**Conclusions:**

LDOC1-mediated chromatin remodeling, through regulation of H2Bub1 recruitment and turnover, represents a key mechanism promoting NSCLC progression. The LDOC1–H2Bub1 axis, potentially involving THAP12, shapes chromatin accessibility and metastatic transcriptional programs, providing mechanistic and clinical insights into tumor aggressiveness and therapeutic response.

**Supplementary Information:**

The online version contains supplementary material available at 10.1186/s12964-025-02607-z.

## Abstract (plain language summary)

Non-small cell lung cancer (NSCLC) is one of the most common and deadly forms of cancer. In this study, we explored the role of a protein called LDOC1 in NSCLC and how it works together with another protein, THAP12. We found that LDOC1 can bind to histone proteins—molecules that help package DNA in the cell nucleus—and works with another protein to break down a modified form of histone H2B called H2Bub1. In this way, LDOC1 normally helps keep H2Bub1 levels under control. THAP12 appears to help bring LDOC1 to chromatin in the nucleus, strengthening this regulatory effect.

When LDOC1 was lost in NSCLC cells, the total amount of H2Bub1 in the cell increased, but its proper distribution on DNA was disrupted. This led to DNA becoming more compact and altered the activity of many genes that control how cancer cells change shape, move, and spread. Without LDOC1, NSCLC cancer cells more easily switch between different shapes (epithelial and mesenchymal states) and adopt a flexible “in-between” state that is linked to greater invasiveness.

In tumor samples from patients, high H2Bub1 levels were linked to areas where cancer had spread into nearby air spaces in the lung (a pattern called spread through air spaces, or STAS) and were associated with worse outcomes for patients receiving chemotherapy. Our findings suggest that LDOC1 and H2Bub1 could be used together as biomarkers to identify aggressive lung cancers and guide treatment choices, as well as provide new targets for therapy.

**Supplementary Information:**

The online version contains supplementary material available at 10.1186/s12964-025-02607-z.

## Background

Lung cancer remains the leading cause of cancer-related death worldwide and is classified as non-small cell lung cancer (NSCLC) or small cell lung cancer (SCLC). NSCLC accounts for approximately 85% of cases and includes subtypes such as adenocarcinoma, squamous cell carcinoma, and large cell carcinoma [1]. Its subtype distribution varies geographically, with adenocarcinoma being more prevalent in East Asian and nonsmoking populations, whereas squamous cell carcinoma predominates in smokers and Western populations [2]. NSCLC exhibits a diverse array of genetic alterations, including EGFR mutations, which are common in East Asian patients; KRAS mutations, which are prevalent in smokers in Western countries; and ALK and ROS1 rearrangements [3–6]. In contrast, SCLC is characterized by rapid growth, early dissemination, and limited genetic diversity, driven mainly by TP53 and RB1 inactivation [7]. These differences reflect both histological and molecular diversity in lung cancer. In addition to genetic alterations, epigenetic dysregulation critically contributes to lung cancer pathogenesis [8]. Among histone posttranslational modifications such as acetylation, methylation, and ubiquitination, monoubiquitination of histone H2B at lysine 120 (H2Bub1) stands out for its substantial impact on chromatin structure [9]. H2Bub1 is primarily catalyzed by the RNF20/RNF40 E3 ubiquitin ligase complex and cooperating E2 enzymes, and is removed by several deubiquitinases, including USP22 and other components of the SAGA deubiquitinase module [10]. Thus, cellular H2Bub1 abundance reflects the dynamic balance between these ligases, deubiquitinases, and downstream proteasome-dependent turnover. Functionally, H2Bub1 acts as a master regulator of transcription, facilitating initiation and elongation by loosening chromatin and enhancing DNA accessibility [11–13]. It also promotes crosstalk with H3K4 and H3K79 methylation, establishing transcriptionally active chromatin [14]. Loss of H2Bub1 disrupts chromatin compaction, activates oncogenic immune pathways (e.g., IL-6), impairs the DNA damage response, and correlates with poor differentiation, stemness, and an aggressive phenotype in NSCLC [13, 15, 16]. These findings highlight H2Bub1 as a potential therapeutic target.

Leucine zipper downregulated in cancer 1 (LDOC1) is an X-linked gene encoding a 146-amino acid protein originally identified as downregulated in cancer cell lines [17]. Structurally, LDOC1 contains a leucine zipper motif that mediates dimerization and a proline-rich SH3-like domain that is implicated in signal transduction. Although LDOC1 lacks a canonical nuclear localization signal, it predominantly resides in the nucleus. It also features adaptor-binding motifs [18] and a highly acidic C-terminal tail [residues 132–145 (DDDEDDDDEEEEDD)] (Additional file 1), suggesting potential interactions with basic proteins such as histones and implicating it in chromatin-associated functions. The potential epigenetic function of LDOC1 was supported by transcriptome profiling of LDOC1-deficient cell lines—including A549 (GSE235829) and PC9 (GSE298765)—which revealed widespread alterations in gene expression, indicating that LDOC1 broadly influences transcriptional programs (Additional file 2). Additionally, our group has shown that LDOC1 is frequently silenced by promoter hypermethylation in smoking-associated OSCC and that its suppression by cigarette smoke contributes to neoplastic transformation [19, 20]. In NSCLC, LDOC1 has different effects depending on the tumor type: in EGFR-mutant (EGFR^M^) tumors, it suppresses tumor growth by modulating EGFR trafficking and downstream RTK signaling via interactions with AP1 and AP2 adaptor complexes [18], whereas in EGFR-wild-type (EGFR^WT^) NSCLC, it promotes tumorigenesis by sustaining IL-6/JAK2/STAT3 signaling [21]. These observations establish LDOC1 as a multifaceted regulator of oncogenic signaling, endocytic trafficking, and potentially chromatin remodeling.

This study aimed to elucidate the epigenetic role of LDOC1 in NSCLC. Using LDOC1-deficient and LDOC1-expressing EGFRᵂᵀ NSCLC cell lines and clinical samples, we show that LDOC1 loss disrupts genome-wide H2Bub1 recruitment, drives chromatin remodeling, and alters cytoskeleton-related gene expression, increasing metastatic potential. Furthermore, we identify H2Bub1 as a prognostic biomarker in NSCLC patients receiving chemotherapy and reveal a complex interactome involving LDOC1, H2B, H2Bub1, and related factors, underscoring its epigenetic function and tumor-suppressive role in EGFRᵂᵀ NSCLC.

## Methods

### Cell culture

A549, H1299, and H460 NSCLC cells were maintained in RPMI-1640 medium supplemented with 10% FBS and 1% penicillin–streptomycin at 37 °C in a humidified 5% CO₂ incubator. LDOC1 knockdown or overexpression sublines were established and maintained under standard selection and passaging conditions as described in our previous studies [21]. All cell lines were regularly tested for mycoplasma contamination and passaged at sub-confluent densities to ensure genomic stability.

### Reagents

The following antibodies were used: rabbit polyclonal anti-LDOC1 (custom-made); anti-H2B (Abcam, ab1790; WB, IP, IF); anti-H2Bub1 (ubiquityl-Histone H2B [Lys120], Cell Signaling Technology, #5546; WB, IF, ChIP); anti-GAPDH (GeneTex, GTX100118) and anti-THAP12 (Bethyl, A300-586 A; WB, IF, IP); anti-PSMA1 (Abnova, H00005682-M01; WB, IF, IP); FITC-conjugated anti–α-tubulin (Sigma-Aldrich, F2168; IF); anti-ubiquitin (Cell Signaling Technology, #3936; WB); and anti–E-cadherin (Cell Signaling Technology, #3195; WB, IF). Antibodies for flow cytometry included APC anti-human CD324 (E-Cadherin) (BioLegend, #324107), PE anti-vimentin (BioLegend, #677804), and isotype controls (BioLegend, #401209, #400121, #400113, #400507). Chemical reagents included recombinant human TGF-β (MCE, HY-P7118), the proteasome inhibitor bortezomib (MCE, HY-10227) and recombinant human EGF (MCE, HY-P7109).

### Preparation of histone extracts

Histone proteins were extracted from cultured cells using an acid extraction protocol as previously described [22]. Briefly, cells were harvested and washed twice with ice-cold PBS, then resuspended in hypotonic lysis buffer and incubated on ice to isolate nuclei. The nuclear pellet was collected by centrifugation and extracted with 0.2 N HCl overnight at 4 °C with gentle agitation. Acid-soluble proteins were recovered by centrifugation, and the supernatant containing histones was neutralized with 1 N NaOH before downstream analyses. Protein concentration was determined using the Bradford assay.

### Proximity ligation assay (PLA) fluorescence staining

In situ protein–protein interactions involving LDOC1 were detected using the Duolink^®^ In Situ Red Starter Kit Mouse/Rabbit (Sigma-Aldrich, Cat. No. DUO92101) or Duolink^®^ In Situ Detection Reagents Green (Sigma-Aldrich, Cat. No. DUO92013), following the manufacturer’s instructions. Briefly, cells grown on coverslips were fixed with 4% paraformaldehyde for 15 min, permeabilized with 0.1% Triton X-100 for 10 min, and blocked in Duolink blocking buffer. Cells were then incubated overnight at 4 °C with primary antibodies against LDOC1 and its interaction partners, including PSMA1, H2B, H2Bub1, and THAP12. After incubation with PLA probes and ligation, PLA signals were amplified and visualized according to the kit protocol. Nuclei were counterstained with DAPI. Images were acquired using a Leica Stellaris 8 confocal microscope and processed with LAS X software.

### Coimmunoprecipitation (CoIP) and western blotting

Cells were lysed in ice-cold IP lysis buffer (Pierce, Thermo Fisher Scientific, IL, USA) and clarified by centrifugation. Equal amounts of protein (LDOC1–PSMA1, LDOC1–THAP12) or protein plus acid-extracted histones (H2B/H2Bub1) were pre-cleared with control IgG and protein A/G magnetic beads (TOOLS Biotechnology, Taipei, Taiwan), incubated with primary antibodies or control IgG for 2 h at 4 °C, and then with protein A/G magnetic beads overnight at 4 °C. Complexes were washed, eluted in SDS sample buffer, and analyzed by immunoblotting. For H2B/H2Bub1 IPs, A549-shCtrl and A549-shLDOC1 cells were with or without pretreatment of bortezomib (10 nM, 20 min) before lysis.

### Chromatin Immunoprecipitation with sequencing (ChIP-seq) and data analysis

ChIP-seq was performed as described previously [23] with minor modifications. Cells were crosslinked with 1% formaldehyde for 10 min at room temperature, quenched with 125 mM glycine, washed with ice-cold PBS, lysed, and sonicated (Bioruptor Plus, Diagenode) to ~ 500 bp fragments. Sheared chromatin was incubated overnight at 4 °C with anti-H2Bub1 antibody (Cell Signaling Technology, #5546) or control IgG and Protein A/G magnetic beads. After sequential washes, DNA–protein complexes were eluted, reverse crosslinked at 65 °C overnight, and DNA was purified for library preparation and sequencing (Taiwan Genomic Industry Alliance Inc., Taipei, Taiwan). Reads were quality-checked, aligned to hg38 using Bowtie2, and peaks called with MACS3 (v3.0.1). Genome browser tracks were visualized in IGV and UCSC Genome Browser, and peak annotation/distribution analyses were performed with ChIPseeker (v1.44.0, R/Bioconductor) [24, 25].

### Micrococcal nuclease (MNase) digestion assay

Chromatin compaction was assessed by MNase digestion as described previously [26]. Briefly, ~ 1 × 10⁶ cells were washed with cold PBS, resuspended in hypotonic buffer (10 mM Tris-HCl, pH 7.5; 10 mM NaCl; 3 mM MgCl₂; 0.5% NP-40), and nuclei pelleted. Nuclei were resuspended in MNase digestion buffer (10 mM Tris-HCl, pH 7.5; 15 mM NaCl; 60 mM KCl; 1 mM CaCl₂) and digested with 1 U MNase (New England Biolabs) at 37 °C for the indicated times. Reactions were stopped with 10 mM EDTA, DNA purified by phenol–chloroform extraction, and analyzed by 1.5% agarose gel electrophoresis to visualize nucleosome patterns.

### Assay for transposase-accessible chromatin with sequencing (ATAC-seq) and data analysis

 Library preparation and sequencing for ATAC-seq were performed on 1 × 10⁶ cells as described previously [27] with minor modifications. Cells were washed with ice-cold PBS, lysed in ATAC lysis buffer (10 mM Tris-HCl, pH 7.4; 10 mM NaCl; 3 mM MgCl₂; 0.1% NP-40; 0.1% Tween-20), and nuclei isolated by sequential detergent treatment and centrifugation. Nuclei were resuspended in 50 µl transposition mix (Illumina TDE1 transposase) and incubated at 37 °C for 30 min at 300 rpm. DNA fragments were purified (MinElute PCR Purification Kit, Qiagen), PCR-amplified (NEBNext PCR Master Mix, New England Biolabs) with cycle number determined by qPCR, and libraries quality-checked before paired-end sequencing on an Illumina NextSeq 550. ATAC-seq data were processed and analyzed using the same pipeline as for ChIP-seq.

### Gene expression microarray and quantitative reverse transcription–polymerase chain reaction (qRT–PCR)

Total RNA was extracted using the RNeasy Mini Kit (Qiagen) and its quality assessed with a BioAnalyzer (Agilent Technologies, CA, USA). Transcriptome profiling was carried out using the HT06-Clariom™ S Assay Human platform (Thermo Fisher Scientific, MA, USA) at the Microarray Core Facility, NHRI, Taiwan. Differential expression analysis was performed with Transcriptome Analysis Console (TAC) software (Thermo Fisher Scientific), and pathway enrichment analysis of differentially expressed genes (DEGs) was conducted using MetaCore software (Clarivate, London, UK). For qRT–PCR validation, reverse transcription quantitative PCR (RT-qPCR) was performed using the Power SYBR™ Green PCR Master Mix (Applied Biosystems, CA, USA) with gene-specific primers (Additional file 3). All RT-qPCR reactions were performed in triplicate using an ABI PRISM 7000 Sequence Detection System (Applied Biosystems).

### Wound healing migration assay

Cell migration was assessed using the Incucyte^®^ Scratch Wound Assay (Sartorius) following the manufacturer’s instructions. A549 and H460 cells were pretreated ± TGF-β (10 ng/mL) for 2 days, seeded into 96-well ImageLock plates (2 × 10⁴ and 4 × 10⁴ cells/well, respectively), and grown to confluence. Scratch wounds were generated with the Incucyte^®^ 96-Well WoundMaker, washed with PBS, and cultured in medium ± TGF-β (10 ng/mL) at 37 °C in an Incucyte^®^ Live-Cell Analysis System. Images were acquired every 2 h for 48 h, and wound closure was quantified as relative wound density using Incucyte^®^ software.

### Adhesion assay

Cells were cultured ± TGF-β (10 ng/mL) for 2 days, serum-starved overnight, detached with 10 mM EDTA for 10–15 min, washed with DMEM, and resuspended in DMEM containing 0.1% BSA. Cells (1 × 10⁵/well) were seeded onto collagen I-coated 96-well plates and incubated at 37 °C for 40 min. Non-adherent cells were removed by four DMEM washes, and adherent cells were allowed to recover in DMEM with 10% FBS for 4 h. Wells were washed with PBS, fixed in 4% paraformaldehyde, stained with 0.1% crystal violet, and absorbance measured at 590 nm.

### Immunofluorescence staining of microtubules

Cells grown on coverslips ± TGF-β (10 ng/mL) were fixed with 4% paraformaldehyde for 15 min, permeabilized with 0.1% Triton X-100/PBS for 10 min, and blocked with 1% BSA/PBS for 30 min. Samples were incubated with FITC-conjugated anti–α-tubulin (1:500; Sigma-Aldrich, F2168) for 1 h at room temperature in the dark, washed, and counterstained with DAPI. Coverslips were mounted with ProLong™ Diamond Antifade Mountant (Invitrogen) and imaged on a Leica Stellaris 8 confocal microscope using LAS X software.

### Flow cytometry

Cells were trypsinized, neutralized with complete medium, collected by centrifugation (1500 rpm, 5 min, 4 °C), washed twice with PBS, and stained in cold PBS with APC–E-cadherin and PE–vimentin antibodies for 20 min at 4 °C in the dark. After washing, cells were passed through a 40 μm strainer (Falcon™, Corning) and analyzed on an Attune™ NxT Flow Cytometer (Thermo Fisher Scientific). Data were processed with FlowJo™ v10.10.1 (BD Life Sciences), and epithelial–mesenchymal hybrid cells were identified by marker co-expression.

### Clinical samples and immunohistochemistry (IHC)

Tumor specimens and clinical data were obtained from 127 patients with EGFRᵂᵀ NSCLC who underwent bronchoscopic biopsy, transthoracic biopsy, or surgery at National Taiwan University Hospital Hsin-Chu Branch (2012–2021), with follow-up periods of 0.2–75.5 months. Clinicopathological characteristics and chemotherapy status are summarized in Table 1. H&E-stained sections were reviewed and classified as adenocarcinoma or non-small cell carcinoma according to 2021 WHO criteria. The study was approved by the Research Ethics Committee B of National Taiwan University Hospital (202303103RINB and 111-171-F) and the National Health Research Institutes (EC1130404). FFPE Sections (5 μm) were deparaffinized, rehydrated, and subjected to antigen retrieval in citrate buffer (pH 6.0, 30 min), followed by blocking with 0.5% H₂O₂ in methanol. Sections were incubated overnight at 4 °C with a custom anti-LDOC1 antibody (1:150) or anti-H2Bub1 antibody (1:200; D11, Cell Signaling), and signals detected using the BOND-PRIME Polymer DAB system. LDOC1 expression was classified as negative (absent/weak) or positive (moderate/strong) cytoplasmic or nuclear–cytoplasmic staining. Nuclear H2Bub1 was quantified by H-score = [1 × (% weak) + 2 × (% moderate) + 3 × (% strong)] (range: 0–300), with low expression defined as H-score < 100. Of the 127 EGFR^WT^ NSCLC cases, 98 had sufficient primary tumor tissue for paired LDOC1/H2Bub1 IHC assessment. For STAS-focused analyses, STAS foci with adequate staining quality were evaluable in 75 cases.

**Table 1:** Clinicopathological characteristics and chemotherapy status of patients whose archived tumor specimens were analyzed

*All patients *(*n* = 127)	No.
Age	
≤ 75	98
> 75	29
*Gender*	
Female	67
Male	60
*Stage*	
I + II	65
III + IV	62
*Smoking status*	
Never	78
Former/current	49
Chemotherapy	
Treated	75
Untreated	52
STAS	
Positive	77
Negative	50

### Bioinformatic and statistical analysis

TCGA LUAD datasets were obtained from UCSC Xena (https://xena.ucsc.edu/). Associations between H2Bub1 expression and clinicopathological parameters were assessed using chi-square or Fisher’s exact tests. McNemar’s test was applied to paired categorical data from matched primary tumors and corresponding STAS lesions. Continuous variables were compared using Student’s t-test or Mann–Whitney U test, and group differences with Welch’s t-test. *p* < 0.05 was considered statistically significant. Kaplan–Meier survival analysis with the log-rank test was used to estimate survival probabilities.

## Results

### LDOC1 modulates H2Bub1 abundance and histone turnover through a proteasome-linked interactome

**Fig. 1:**
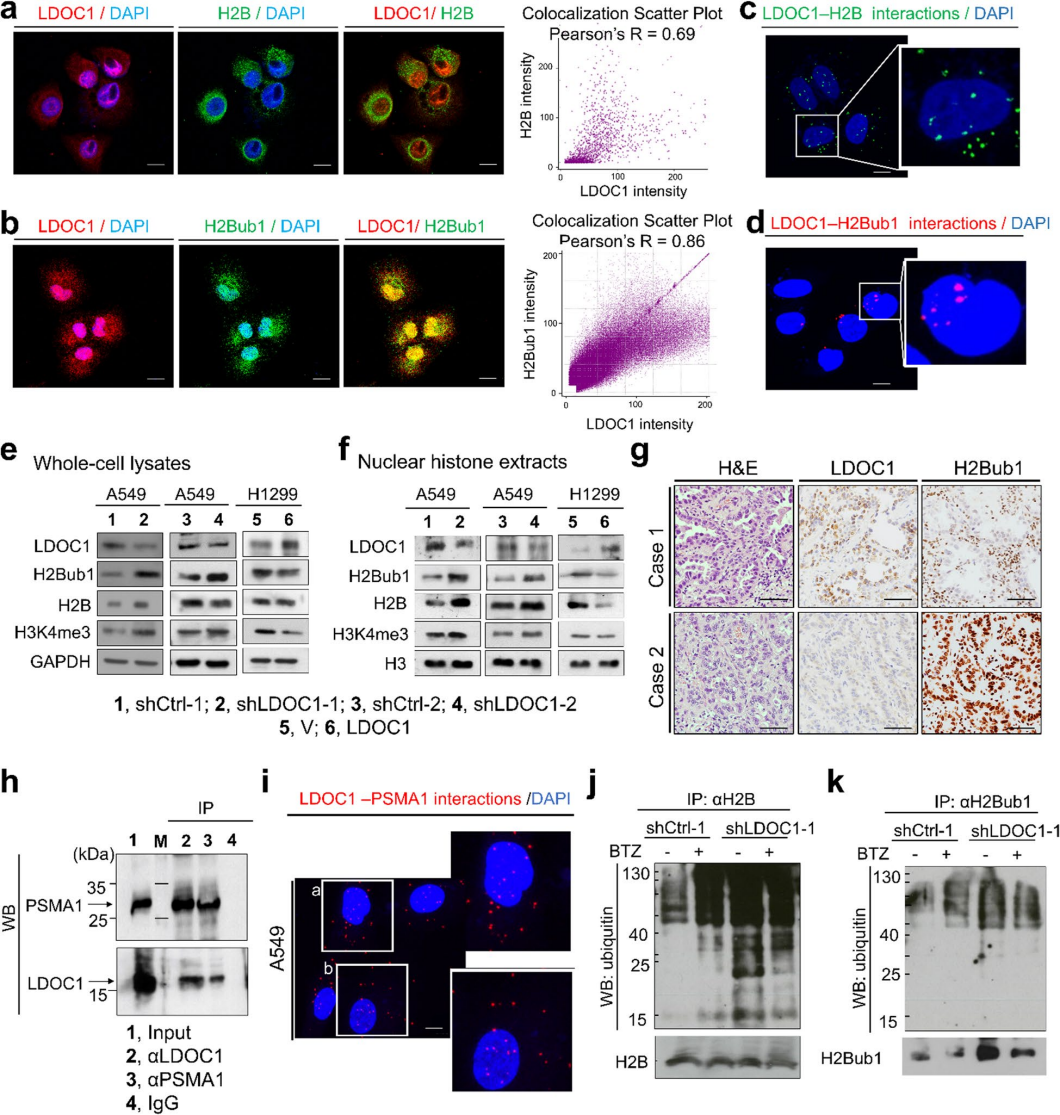
LDOC1 regulates H2Bub1 abundance and histone turnover through interactions with H2B/H2Bub1 and the proteasome.** a–b** Confocal immunofluorescence of A549 cells showing LDOC1 (red) colocalized with H2B (green, a) or H2Bub1 (green, b); nuclei are counterstained with DAPI (blue). Right, colocalization scatter plots with Pearson’s correlation coefficient (R). **c–d** PLA detecting in situ interactions between LDOC1–H2B (c, green puncta) or H2Bub1 (d, red puncta) in A549 cells; nuclei: DAPI. **e–f** Immunoblotting of whole-cell lysates (**e**) and nuclear histone extracts (**f**) from A549 sublines with LDOC1 knockdown (shLDOC1-1/-2) and matched controls, and from H1299 cells with ectopic LDOC1 expression. GAPDH (whole-cell lysates) and histone H3 (histone extracts) served as loading controls; for histone extracts, equal cell numbers were processed to ensure comparable loading. **g** Representative H&E and IHC staining for LDOC1 and H2Bub1 in two lung adenocarcinoma cases. **h** Co-immunoprecipitation (CoIP) followed by WB showing association between LDOC1 and PSMA1 in A549 cells; input and IgG controls are shown. **i** PLA signals indicating LDOC1–PSMA1 interactions in A549 cells; nuclei, DAPI. **j–k** IP of H2B (**j**) or H2Bub1 (**k**) from A549 cells with or without LDOC1 knockdown and with or without BTZ (20 nM, 30 min), followed by WB to detect polyubiquitinated species. Whole-cell lysates combined with histone extracts were precleared with control IgG and immunoprecipitated with the indicated antibodies; immunoprecipitates were probed with anti-ubiquitin, anti-H2B, and anti-H2Bub1. Microscopy: Panels a–d and i were acquired on a Leica Stellaris 8 confocal microscope at 400**×**; scale bars, 10 μm

Given the importance of H2Bub1 in NSCLC, we first assessed the effect of LDOC1 on this histone modification. In A549 cells, double immunofluorescence staining revealed strong nuclear colocalization of LDOC1 with H2B (Pearson’s *r* = 0.69) and with H2Bub1 (Pearson’s *r* = 0.86), as confirmed by intensity–scatter plots (Fig. 1a, b). PLA further verified in situ interactions of LDOC1 with both H2B and H2Bub1 as discrete nuclear foci. (Fig. 1c, d). To probe function, we generated two LDOC1 − depleted A549 sublines (shLDOC1 − 1 and shLDOC1 − 2) using independent shRNAs. Immunoblotting of whole-cell lysates and nuclear histone extracts showed that LDOC1 loss elevated H2Bub1, H2B, and H3K4me3—a downstream histone mark of H2Bub1—whereas ectopic LDOC1 expression in H1299 cells decreased H2Bub1 and H3K4me3 without altering total H2B in whole-cell lysates (Fig. 1e, f). Consistent with these cell-based findings, IHC analysis of advanced EGFRᵂᵀ NSCLC tumor samples (*n* = 98) revealed a significant association between LDOC1 and H2Bub1: 84% (36/43) of LDOC1-low tumors exhibited high H2Bub1, versus 64% (35/55) of LDOC1-high tumors; 36% (20/55) of LDOC1-high cases displayed reduced H2Bub1 (*P* = 0.027) (Table 2, Fig. 1g). To explore a proteasome-linked mechanism, mining of IntAct database identified proteasome subunit alpha type 1 (PSMA1), which has been reported to exert substrate-specific deubiquitinating activity and oncogenic functions [28], as a candidate LDOC1 interactor. Co-immunoprecipitation confirmed LDOC1–PSMA1 association in A549 cells (Fig. 1h), and PLA detected LDOC1–PSMA1 signals in both the nucleus and cytoplasm (Fig. 1i). To determine whether LDOC1 influences H2B ubiquitination and proteasomal turnover, we immunoprecipitated H2B and probed for polyubiquitin. In control cells, treatment with the proteasome inhibitor bortezomib (BTZ) caused accumulation of polyubiquitinated H2B, whereas A549-shLDOC1-1 cells exhibited elevated polyubiquitination even in the absence of BTZ, with only a modest further increase upon BTZ treatment (Fig. 1j). Similarly, immunoprecipitation with anti-H2Bub1 antibodies revealed higher levels of polyubiquitinated H2Bub1 in LDOC1-deficient cells irrespective of proteasome inhibition (Fig. 1k). Together, these findings support a model in which LDOC1 restrains the accumulation of H2B and H2Bub1 by promoting their proteasome-dependent turnover, potentially through interaction with PSMA1, thereby helping to maintain histone homeostasis.

**Table 2:** Association between LDOC1 and H2Bub1 protein expression in primary tumors from EGFRᵂᵀ NSCLC patients

*All patients *(*n* = 98)	H2Bub1		
	Low expression	High expression	
*LDOC1*			*P* value
Low expression	7	36	0.027
High expression	20	35	

### LDOC1 knockdown leads to widespread loss of H2Bub1 chromatin occupancy

**Fig. 2:**
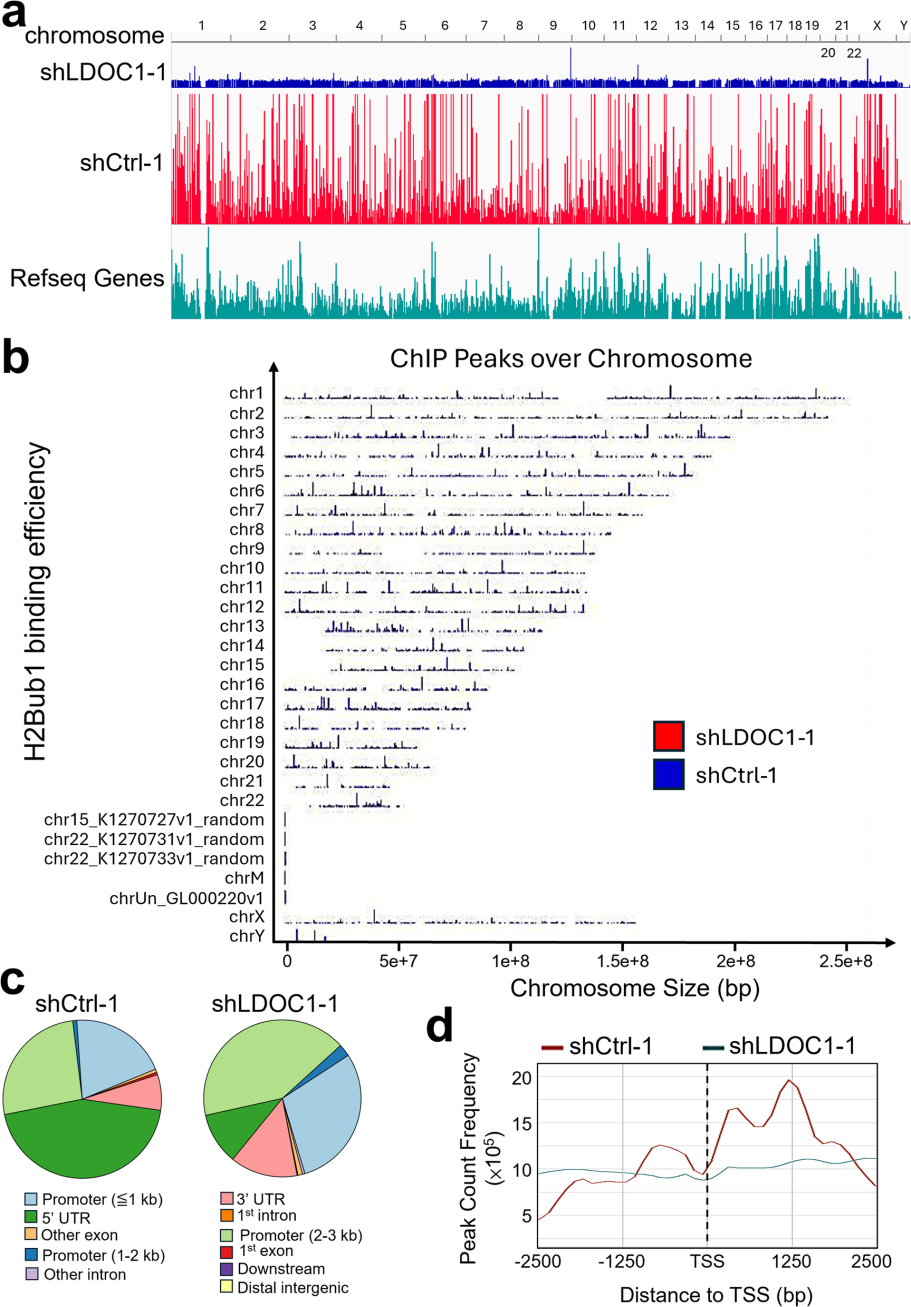
Genome-wide profiling of H2Bub1 distribution by ChIP-seq in A549 cells.** a** Genome browser view of H2Bub1 ChIP-seq signals across all chromosomes in A549-shCtrl-1 and A549-shLDOC1-1 cells, aligned with RefSeq gene annotations. **b** Chromosomal distribution of H2Bub1 ChIP peaks across the genome in A549-shCtrl-1 and A549-shLDOC1-1 cells. Peak densities are visualized relative to chromosome size. **c** Genomic annotation of H2Bub1-enriched regions based on gene features, including promoter, exon, UTR, intron, and intergenic regions. Pie charts showing the relative percentage of H2Bub1 peaks assigned to each category in A549-shCtrl-1 and A549-shLDOC1-1 cells. **d** Metagene analysis of H2Bub1 occupancy across ± 2.5 kb surrounding the transcription start site (TSS) in A549-shCtrl-1and A549-shLDOC1-1 cells

To investigate how LDOC1 influences the genomic distribution of H2Bub1, we performed ChIP–seq using an anti-H2Bub1 antibody in A549-shCtrl-1 and A549-shLDOC1-1 cells. Genome browser tracks revealed a pervasive loss of H2Bub1 signal across nearly all chromosomes in LDOC1-depleted cells, despite the elevated global H2Bub1 levels detected by immunoblotting (Fig. 2a, b). Peak annotation in control cells showed that H2Bub1 was strongly enriched at gene-associated regions, with the majority of peaks mapping to the first exon (44.6%) and 3′ UTR (26.2%). Upon LDOC1 knockdown, the fraction of peaks at first exons decreased to 10.7%, whereas peaks at 3′ UTRs increased to 41.7%, together with a relative gain in promoter-proximal regions (Fig. 2c). Metagene profiling further demonstrated that H2Bub1 was enriched around transcription start sites (TSSs) in control cells, but this enrichment was largely lost upon LDOC1 depletion (Fig. 2d), indicating a global reduction of H2Bub1 at active promoters. To validate these findings, we repeated the H2Bub1 ChIP–seq experiment in an independent shRNA pair (A549-shCtrl-2/shLDOC1-2) using a different sequencing platform that generated shorter single-end reads. This second dataset again showed a pronounced decrease in H2Bub1 chromatin enrichment in LDOC1-deficient cells, with far fewer peaks detected in shLDOC1-2 than in shCtrl-2 (4,277 vs. 30,734 peaks; Additional file 4a–c). Peak annotation of this replicate experiment (Additional file 4b, c) likewise demonstrated that H2Bub1 peaks were concentrated in gene-associated regions, although the precise distribution across promoters, exons, and introns differed somewhat from the first dataset. Cross-experiment comparison of log₂ peak intensities further confirmed good reproducibility of H2Bub1 signal between control samples (Pearson *r* = 0.688, *n* = 20,570 shared peaks) and a consistent downward shift in LDOC1-depleted samples (*r* = 0.375, *n* = 3,957 peaks; Additional file 4d, e). Although the detailed distributions of peaks across genomic annotations differed between experiments—likely reflecting differences in sequencing depth, read length, and peak-calling parameters—the overarching pattern was the same: LDOC1 loss leads to a genome-wide reduction of H2Bub1 occupancy at gene-associated regions.

### LDOC1 may facilitate chromatin accessibility through THAP12-dependent H2Bub1 recruitment

**Fig. 3:**
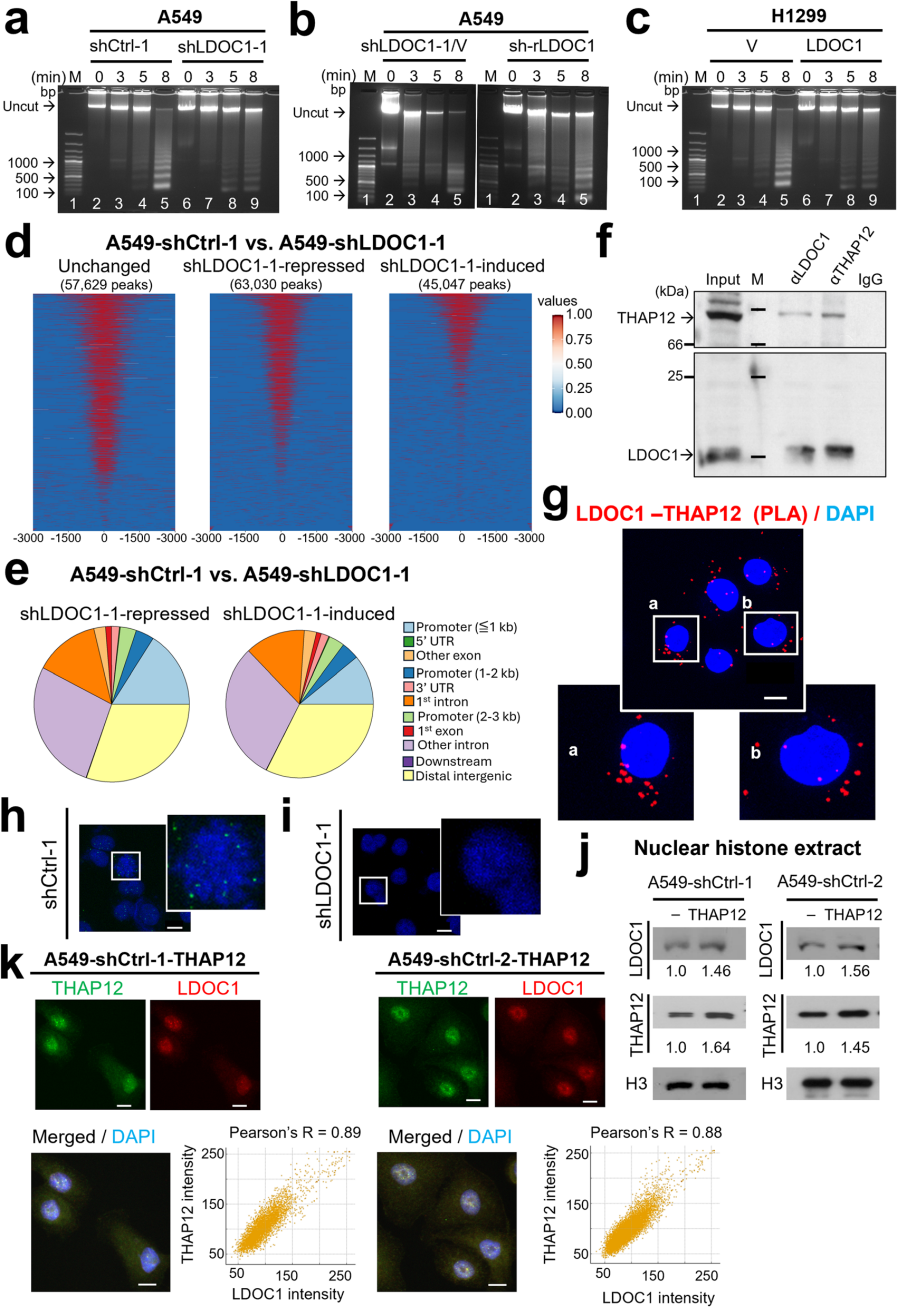
LDOC1 regulates chromatin accessibility and associates with THAP12 in the nucleus.** a-c** MNase digestion of nuclear chromatin from A549-shCtrl-1 and A549-shLDOC1-1 cells (**a)**, A549-shLDOC1-1 cells expressing control vector (A549-shLDOC1-1/V) or LDOC1 (A549-sh-rLDOC1) (**b)**, and H1299 cells expressing empty vector (H1299-V) or LDOC1 (H1299-LDOC1) (**c)**, resolved by agarose gel electrophoresis. **d** Heatmaps of ATAC-seq signal intensity (± 3 kb around peak summits) for unchanged peaks, shLDOC1-1-repressed and shLDOC1-1-induced peaks in A549-shCtrl-1 versus A549-shLDOC1-1 cells. **e** Genomic distribution of each ATAC-seq peaks category annotated by gene-associated features. **f**,** g** LDOC1–THAP12 interaction validated by CoIP/Western blotting (**f**) and PLA (**g)**; insets (a, b) show magnified PLA foci (red). **h**,** i** PLA detection of LDOC1–THAP12 association in A549-shCtrl-1 (**h**) and A549-shLDOC1-1 (**i**) cells; boxed areas are enlarged at right. Scale bars, 10 μm. **j** Immunoblot of nuclear histone extracts from A549-shCtrl-1 and A549-shCtrl-2 cells with or without ectopic THAP12, probed for LDOC1, THAP12, and H3 (loading control); densitometric values shown below lanes represent the mean of two experiments, normalized to the left lane (= 1.0). **k** Confocal images of THAP12 (green) and LDOC1 (red) in A549-shCtrl-1 and A549-shCtrl-2 cells with ectopic THAP12 expression. Nuclei were counterstained with DAPI (blue); right, pixel-intensity colocalization scatter plots of LDOC1 (red channel) versus THAP12 (green channel) with Pearson’s R

Given the established role of H2Bub1 in promoting chromatin relaxation, we first examined whether LDOC1 affects chromatin compaction. Micrococcal nuclease (MNase) digestion assays showed that chromatin from A549-shLDOC1-1 cells was more resistant to nuclease cleavage than chromatin from A549-shCtrl-1 cells, whereas LDOC1 re-expression in A549-shLDOC1-1 cells (A549-sh-rLDOC1) or ectopic LDOC1 expression in H1299 cells restored MNase sensitivity (Fig. 3a–c). These observations are consistent with the reduced H2Bub1 occupancy on chromatin upon LDOC1 knockdown (Fig. 2a–b). To further characterize genome-wide chromatin dynamics, we performed ATAC-seq in A549-shLDOC1-1 and A549-shCtrl-1 cells. This analysis revealed widespread remodeling, with 63,030 regions showing decreased accessibility (shLDOC1-1–repressed peaks) and 45,047 regions showing increased accessibility (shLDOC1-1–induced peaks) in LDOC1-deficient cells (Fig. 3d). Notably, the shLDOC1-1–repressed peaks were more enriched in promoter regions than shLDOC1-1–induced peaks, indicating a preferential loss of accessibility at regulatory elements (Fig. 3e). An independent ATAC-seq experiment using a second shRNA clone (A549-shLDOC1-2 vs. A549-shCtrl-2) yielded a similar pattern, with 47,446 shLDOC1-2–repressed and 5,830 shLDOC1-2–induced peaks among 88,847 unchanged peaks (Additional file 5a). Again, shLDOC1-2–repressed peaks were more enriched in proximal promoter regions (promoter < 1 kb) than shLDOC1-2–induced peaks (Additional file 5b), further supporting the conclusion that LDOC1 depletion predominantly reduces chromatin accessibility at regulatory sites. Because LDOC1 lacks recognizable DNA-binding domain or canonical nuclear localization signals, we next investigated whether a nuclear partner might facilitate its chromatin association. Among putative LDOC1-interacting proteins, THAP12 was prioritized for validation because it emerged as a high-confidence LDOC1 interactor in the IntAct database and is a nuclear THAP-domain factor implicated in transcriptional and chromatin regulation [29], making it a strong candidate mediator of LDOC1–H2Bub1–dependent effects. Co-immunoprecipitation followed by immunoblotting confirmed that endogenous LDOC1 and THAP12 form a complex in A549 cells (Fig. 3f). Proximity ligation assays (PLA) further demonstrated discrete nuclear foci of LDOC1–THAP12 interactions, which were readily detected in A549 cells and reduced upon LDOC1 knockdown (Fig. 3g–i). To test whether THAP12 influences LDOC1 chromatin association, we generated THAP12-overexpressing A549 sublines (Additional file 6) and examined nuclear histone extracts from A549-shCtrl cells with or without THAP12 overexpression. THAP12 overexpression increased both THAP12 and LDOC1 levels in the nuclear histone fraction (Fig. 3j), and confocal imaging revealed extensive nuclear co-localization of LDOC1 and THAP12, with high Pearson’s correlation coefficients (*r* = 0.89 and 0.88 in A549-shCtrl-1 and − 2 cells, respectively; Fig. 3k, l). Together with the LDOC1–H2Bub1 interaction (Fig. 1b, d), these findings support a model in which THAP12 tethers LDOC1 to chromatin, where LDOC1 in turn contributes to proper H2Bub1 localization and maintenance of chromatin accessibility. To evaluate the functional consequence of this interaction, we examined the effect of THAP12 on cell invasion in the presence or absence of LDOC1. THAP12 overexpression almost completely abolished invasion in LDOC1-proficient A549-shCtrl cells (~ 93% reduction), whereas it only partially attenuated the hyper-invasive phenotype of A549-shLDOC1 cells (~ 62% reduction) (Additional file 7). These data indicate that LDOC1 is required for the full anti-invasive activity of THAP12 and, together with the chromatin and colocalization data, further support a model in which THAP12 cooperates with LDOC1 to recruit H2Bub1 to chromatin and epigenetically constrain the invasive potential of A549 cells.

### Integrated transcriptomic and ChIP-seq analysis reveals LDOC1–H2Bub1 axis control of metastasis-related genes

**Fig. 4:**
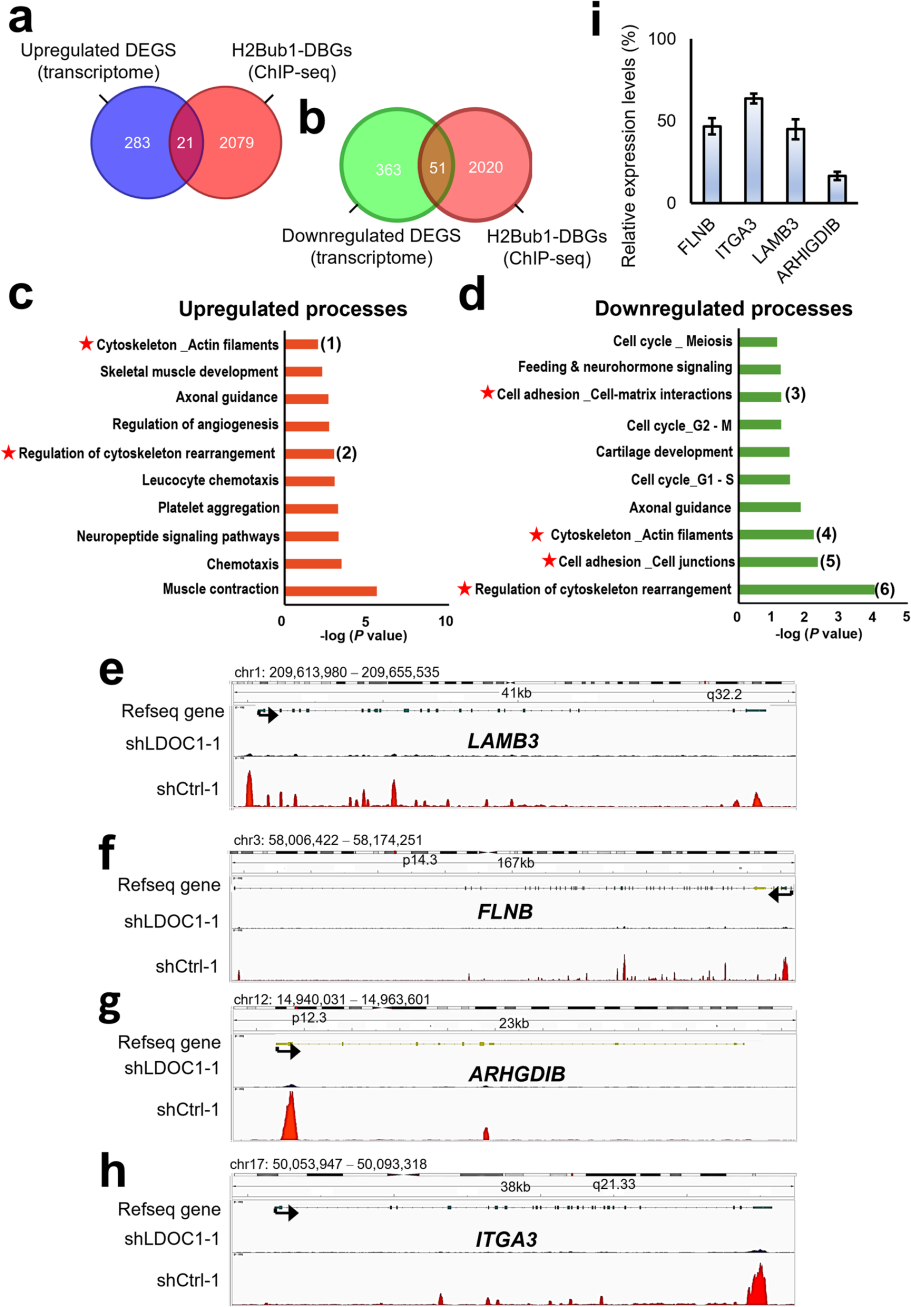
Integrative analysis of ChIP-seq and transcriptomic data identifies metastasis-associated genes regulated by the LDOC1-H2Bub1 axis in A549 cells. **a, b** Venn diagrams showing the overlap between differentially expressed genes (DEGs) from the transcriptomic analysis and H2Bub1-differentially bound genes (DBGs) from the ChIP-seq analysis. (a) Upregulated DEGs (blue) and H2Bub1-DBGs (red); (b) downregulated DEGs (green) and H2Bub1-DBGs (red). **c, d** MetaCore pathway enrichment of overlapping DEGs and H2Bub1-DBGs in A549-shLDOC1 cells, showing the biological processes associated with the upregulated (c) and downregulated (d) genes. Metastasis-related pathways are indicated by red stars, with representative genes as follows: (1) EVL, GNAZ, MYL6, MYL12A; (2) EVL, GNAZ, MYL6, MYL12A; (3) LAMB3, ITGA3, SGCD; (4) SPTBN1, FLNB, SGCD; (5) SFN, SPTBN1, JAM3, PTPN14; (6) SFN, SPTBN1, SGCD, FLNB, ARHGDIB. **e–h** Integrative Genomics Viewer (IGV) tracks showing H2Bub1 ChIP-seq signal intensity at the LAMB3 (e), FLNB (f), ARHGDIB (g), and ITGA3 (h) gene loci in A549-shCtrl-1 and A549-shLDOC1-1 cells. The arrows indicate the direction of transcription. **i** qRT‒PCR validation of FLNB, ITGA3, LAMB3, and ARHGDIB mRNA expression in A549-shCtrl-1 and A549-shLDOC1-1 cells. The expression levels were normalized to those of β-actin. The data are presented as the means ± standard errors of the means (SEMs) from biological replicates.

To investigate the functional consequences of LDOC1 depletion in EGFRᵂᵀ NSCLC cells, we analyzed differentially expressed genes (DEGs) between A549-shLDOC1 cells and control cells (GSE235829). Pathway enrichment analysis revealed that LDOC1 knockdown dysregulated multiple metastasis-related processes, including extracellular matrix (ECM) remodeling, cell–matrix adhesion, epithelial–mesenchymal transition (EMT), and chemotaxis (Additional file 8). Representative DEGs related to cytoskeletal remodeling and ECM regulation are highlighted in Additional file 8c, d. To determine whether these transcriptional changes are epigenetically regulated, we integrated transcriptomic data with H2Bub1 ChIP-seq profiles from LDOC1-knockdown and control A549 cells. Among approximately 2,100 H2Bub1-differentially bound genes (DBGs), 21 overlapped with upregulated transcripts, and 51 overlapped with downregulated transcripts (Fig. 4a, b). Pathway enrichment analysis of these overlapping genes revealed significant enrichment in processes related to metastasis, including cytoskeletal rearrangement, actin filament organization, and cell–matrix interactions (Fig. 4c, d). Key genes such as *EVL*,* GNAZ*,* MYL6*,* SPTBN1*,* FLNB*,* ITGA3*, and *LAMB3* were prominently represented among these overlapping targets. ChIP-seq genome browser views further revealed markedly reduced H2Bub1 occupancy at representative loci, including *ARHGDIB*, *ITGA3*, *FLNB*, and *LAMB3*, following LDOC1 knockdown (Fig. 4e–h). Consistent with this reduction in H2Bub1 enrichment, qRT–PCR analysis confirmed the transcriptional downregulation of these genes in LDOC1-deficient cells (Fig. 4i). Together, these results demonstrate that LDOC1 modulates H2Bub1 chromatin binding and the transcription of metastasis-associated genes, thereby coordinating epigenetic and transcriptional programs that contribute to NSCLC progression.

### LDOC1 regulates TGF-β–driven epithelial–mesenchymal plasticity involving H2Bub1

**Fig. 5:**
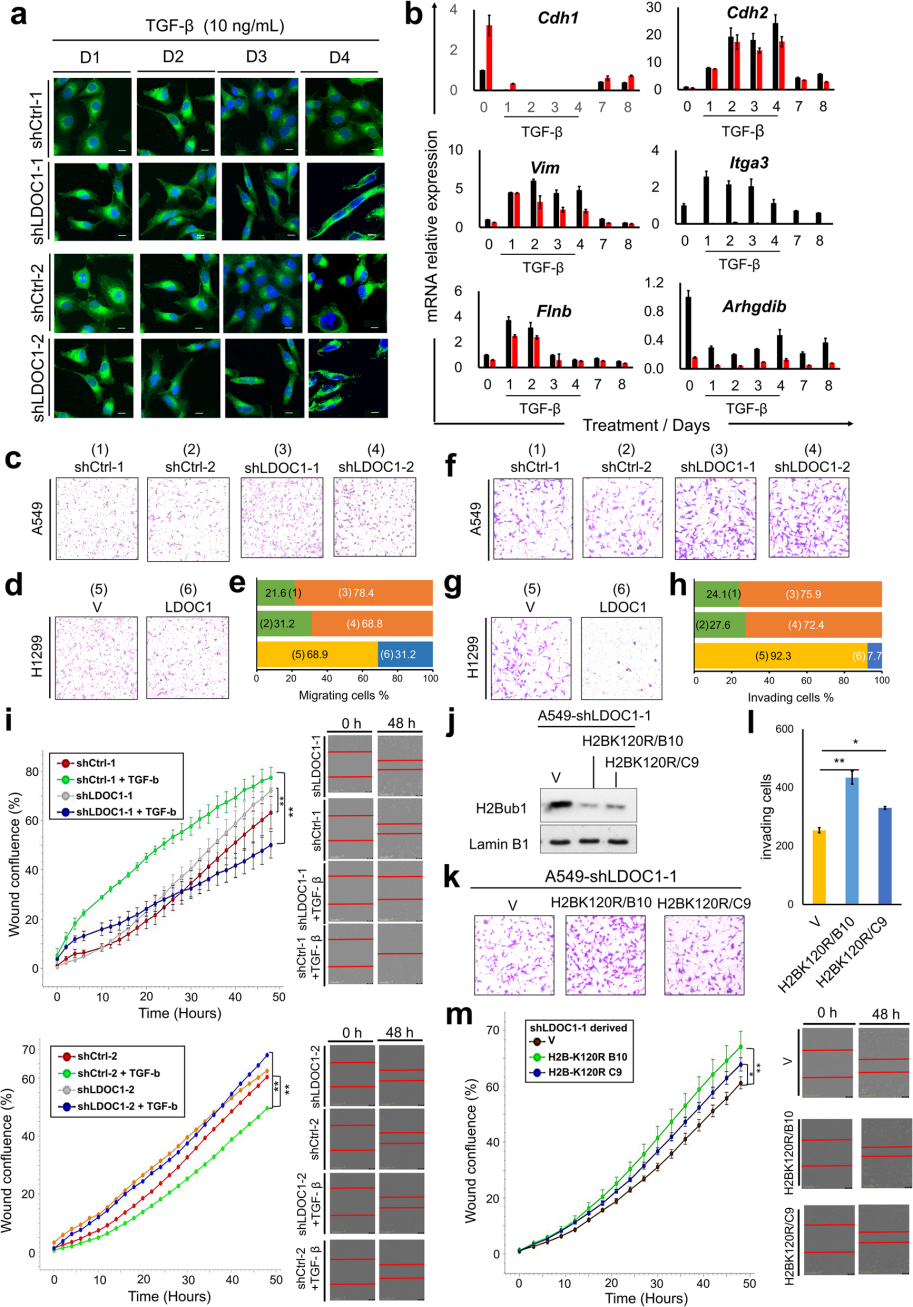
LDOC1 loss alters TGF-β–driven EMT programs and motility through H2Bub1 dysregulation.** a** Confocal images of A549 sublines treated with TGF-β (10 ng/mL) for the indicated days and stained with FITC–anti-α-tubulin (DM1A) and DAPI. Scale bars, 20 μm. **b** Time-course qRT–PCR of EMT- and cytoskeleton-related genes in A549-shCtrl-1 and A549-shLDOC1-1 cells after TGF-β stimulation; expression was normalized to GAPDH (mean ± SEM, *n* = 3). **c–e** Transwell migration of A549-shCtrl-1/-2 and A549-shLDOC1-1/-2 cells (c) and H1299 cells transfected with vector (V) or LDOC1 (**d)**, with quantitative summary in (**e**) (mean ± SEM, *n* = 3). **f–h** Matrigel invasion assays for the same A549 (f) and H1299 (g) panels, with quantification in (h) (mean ± SEM, *n* = 3). **i** Incucyte wound-healing assays showing wound confluence over time in A549-shCtrl and A549-shLDOC1 sublines with or without TGF-β; representative images at 0 h and 48 h are shown. **j** Immunoblot of H2Bub1 in A549-shLDOC1-1 cells expressing H2BK120R mutants (clones B10 and C9); Lamin B1, loading control; V, vector. **k–m** Matrigel invasion (**k**) with quantification (**l**) and wound-healing analysis (m) of A549-shLDOC1-1 cells expressing V or H2BK120R mutants (B10, C9) (mean ± SEM, *n* = 3). *P < 0.05, **P < 0.01 (two-tailed t-test)

Epigenetic mechanisms are central to TGF-β–induced EMT. Because LDOC1 regulates H2Bub1, H3K4me3 and metastasis-associated genes, we next examined its role in TGF-β responses. After stimulation with TGF-β (10 ng/mL), A549-shLDOC1-1 cells rapidly adopted an elongated, mesenchymal morphology by days 3–4, whereas shCtrl-1 cells largely retained a polygonal epithelial appearance; similar changes were observed in the independent shLDOC1-2/shCtrl-2 pair (Fig. 5a). Analysis of EMT and metastasis-related transcripts (Fig. 4) revealed several LDOC1-dependent features (Fig. 5b). LDOC1-deficient cells displayed higher basal CDH1 mRNA despite reduced H2Bub1 occupancy on chromatin. CDH1 declined in both lines upon TGF-β treatment but dropped more sharply in shLDOC1-1 cells before partially recovering after ligand withdrawal. ITGA3 was strongly induced by TGF-β in shCtrl-1 cells yet remained undetectable in shLDOC1-1 cells. FLNB and ARHGDIB were more abundant at baseline in control cells and were downregulated by TGF-β in both genotypes. Functionally, LDOC1 loss markedly enhanced A549 motility: transwell migration increased in shLDOC1-1/-2 cells (78.4%/68.8%) compared with shCtrl-1/-2 cells (21.6%/31.2%), whereas ectopic LDOC1 expression reduced migration in H1299 cells (vector vs. LDOC1: 68.8% vs. 31.2%) (Fig. 5c–e). Transwell invasion assays similarly showed greater invasiveness of A549-shLDOC1-1/-2 cells (75.9%/72.4% vs. 24.1%/27.6% in shCtrl-1/-2) and a pronounced decrease upon LDOC1 overexpression in H1299 cells (vector vs. LDOC1: 92.3% vs. 7.7%) (Fig. 5f–h). Wound-healing assays confirmed increased baseline migration of shLDOC1-1/-2 cells; TGF-β further accelerated migration in shCtrl-1/-2 cells but, intriguingly, suppressed it in LDOC1-deficient cells (Fig. 5i). To test whether these effects involve H2Bub1, we expressed a monoubiquitination-defective H2BK120R mutant (independent clones B10 and C9) in A549-shLDOC1-1 cells. H2BK120R efficiently reduced H2Bub1 levels (Fig. 5j) and significantly increased transwell invasion and, to a lesser extent, wound closure compared with vector controls (Fig. 5k–m). These findings support a model in which LDOC1 constrains TGF-β–driven EMT, adhesion, and migration at least in part through H2Bub1-dependent epigenetic regulation. Taken together, these findings indicate that LDOC1 modulates TGF-β–induced EMT, adhesion and migration, most likely through H2Bub1-dependent epigenetic regulation.

### Hybrid E/M state and elevated H2Bub1 characterize STAS and LDOC1-deficient NSCLC cells, and H2Bub1 has prognostic value in chemotherapy-treated NSCLC patients

**Fig. 6:**
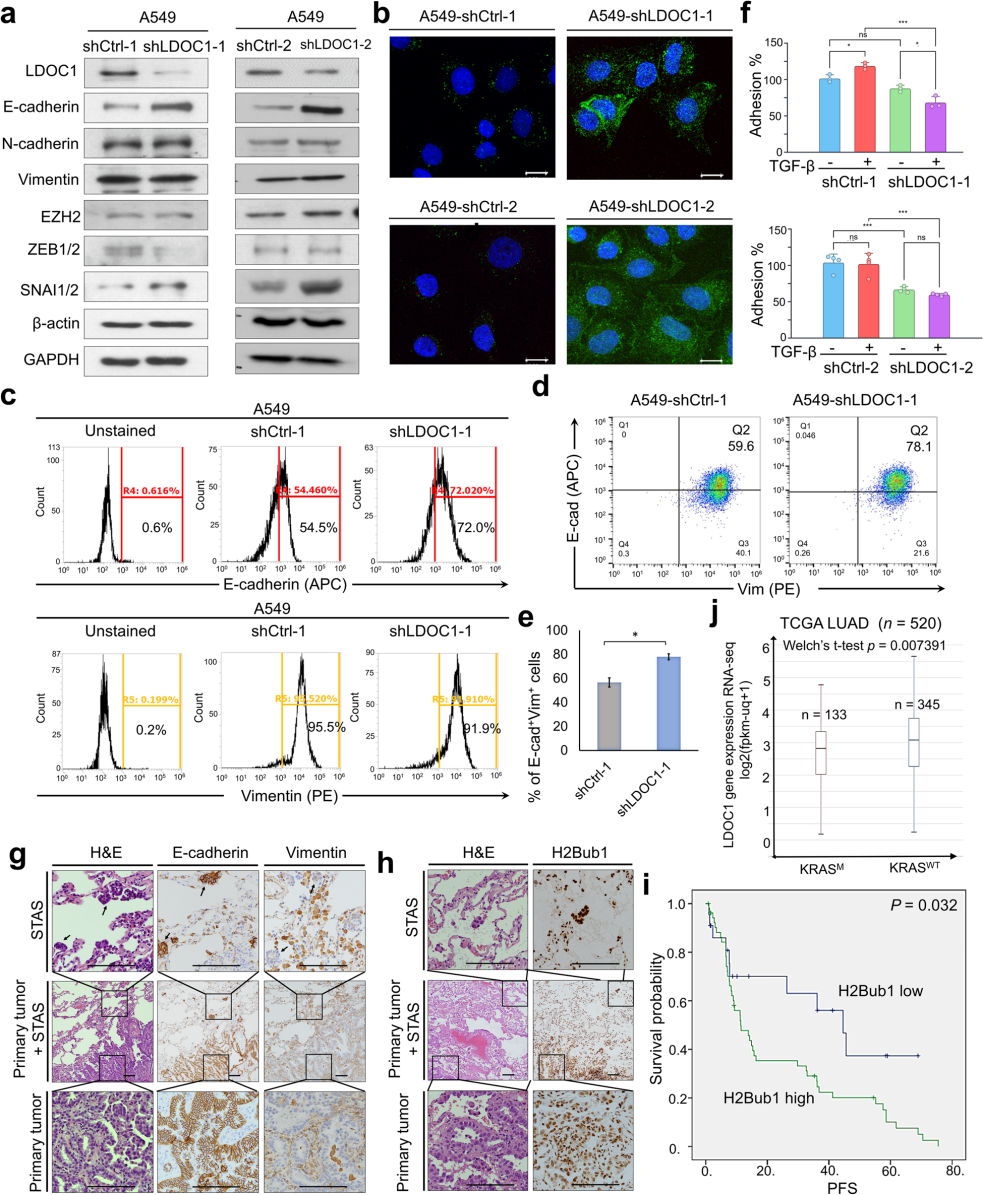
LDOC1 loss promotes a hybrid epithelial–mesenchymal state and elevated H2Bub1 associated with STAS and clinical outcome in NSCLC. a Immunoblot analysis of LDOC1 and EMT markers in A549-shCtrl-1/-2 and A549-shLDOC1-1/-2 cells; GAPDH and β-actin served as loading controls. b Confocal immunofluorescent images of E-cadherin (green) and nuclei (DAPI, blue) in A549 sublines. Scale bars, 10 μm. c–e Flow-cytometric analysis of A549-shCtrl-1 and A549-shLDOC1-1 cells stained for E-cadherin–APC and vimentin–PE: (c) representative single-parameter histograms, (d) bivariate plots showing E-cad⁺/Vim⁻, E-cad⁻/Vim⁺ and E-cad⁺/Vim⁺ populations, and (e) quantification of hybrid E/M (E-cad⁺/Vim⁺) cells (mean ± SEM; *P < 0.05, two-tailed t-test). f Adhesion of A549-shCtrl-1/-2 and A549-shLDOC1-1/-2 cells with or without TGF-β (mean ± SEM, n = 3; *P < 0.05, **P < 0.01, ***P < 0.001, two-tailed t-test). g–h Representative IHC for H&E, E-cadherin and vimentin (g) and H2Bub1 (h) in paired primary tumors and matched STAS lesions from NSCLC; insets show higher magnification. Scale bars, 100 μm. i Kaplan–Meier curves of progression-free survival in chemotherapy-treated NSCLC patients stratified by H2Bub1 expression. j LDOC1 mRNA levels in KRAS-mutant versus KRAS^WT tumors in the TCGA LUAD cohort (n = 520; Welch’s t-test)

As shown in Fig. 5a–b, A549-shLDOC1-1 cells paradoxically maintained high E-cadherin expression despite acquiring a more mesenchymal-like morphology, indicating a decoupling between epithelial marker expression and morphological transformation upon LDOC1 loss. Immunoblotting confirmed that LDOC1 knockdown in two independent sublines (shLDOC1-1/-2) induced a hybrid epithelial–mesenchymal (E/M) program, with co-expression of E-cadherin and mesenchymal markers (N-cadherin, vimentin, EZH2, ZEB1/2, and SNAI1/2), whereas control cells showed lower E-cadherin and SNAI1/2 levels (Fig. 6a). Immunofluorescence revealed stronger and more continuous E-cadherin staining along the plasma membrane in LDOC1-deficient cells (Fig. 6b). Flow cytometry demonstrated an increased fraction of E-cadherin–positive cells in shLDOC1-1 versus shCtrl-1 cells (72.0% vs. 54.5%), while vimentin positivity remained high in both (91.9% vs. 95.5%) (Fig. 6c). Dual E-cadherin/vimentin staining further showed enrichment of hybrid E/M cells in LDOC1-deficient cultures (78.1% vs. 59.6% in shCtrl-1; *P* < 0.05) (Fig. 6d–e). Functionally, LDOC1 knockdown significantly reduced cell adhesion irrespective of TGF-β treatment, whereas TGF-β itself had minimal effect on adhesion in either genotype (Fig. 6f). In EGFR^WT^ NSCLC tumors with STAS lesions (*n* = 75), IHC revealed co-expression of E-cadherin and vimentin in both STAS foci and matched primary tumors, with STAS lesions exhibiting stronger and more extensive staining, indicative of a pronounced hybrid E/M phenotype (Fig. 6g). For H2Bub1 analysis, 38 cases with uniform H2Bub1 staining in STAS lesions were evaluable. H2Bub1 levels were significantly higher in STAS compared with paired primary tumors (*P* = 0.006; Table 3; Fig. 6h), and low LDOC1 expression was strongly associated with increased H2Bub1 in STAS lesions (*P* = 3.56 × 10⁻⁵; Table 4). Among 75 EGFRᵂᵀ NSCLC patients receiving chemotherapy, high H2Bub1 expression was significantly associated with shorter progression-free survival (PFS; *P* = 0.032; median 11.3 vs. 44.8 months for high vs. low H2Bub1; Fig. 6i). Overall survival (OS) showed a similar but non-significant trend (*P* = 0.110; median 11.5 vs. 44.6 months; Additional file 9). LDOC1 protein levels inversely correlated with H2Bub1 (*P* = 0.049, data not shown), although LDOC1 alone was not significantly associated with PFS or OS. Analysis of TCGA LUAD (*n* = 520) further showed that LDOC1 mRNA expression was significantly lower in KRAS-mutant tumors than in KRAS^WT^ tumors (*P* = 0.0074; Fig. 6j). Together, these data indicate that LDOC1 depletion drives a hybrid E/M state and elevated H2Bub1 in A549 cells, mirroring the phenotype of STAS-positive tumor cells, and support H2Bub1 as a potential prognostic biomarker in chemotherapy-treated NSCLC.

**Table 3:** Comparison of H2Bub1 protein expression between STAS lesions and matched primary tumors in EGFRᵂᵀ NSCLC patients

*All patients (*n* = 38)	H2Bub1		
	High expression	Low expression	
STAS	27	11	*P* value
Primary tumor	15	23	0.006

^*^Only primary tumors with surrounding STAS lesions exhibiting uniformly detectable H2Bub1 expression were included in this analysis

**Table 4:** Association between LDOC1 and H2Bub1 protein expression in STAS lesions from EGFR^WT^ NSCLC patients

*All patients *(*n* = 75)	H2Bub1		
	Low expression	High expression	
*LDOC1*			*P* value
Low expression	5	38	3.56 × 10⁻⁵
High expression	19	13	

## Discussion

In this study, we identify LDOC1 as a chromatin-associated tumor suppressor in EGFR^WT^ NSCLC that regulates H2Bub1 abundance, chromatin recruitment, and histone turnover through a complex interactome involving core histones, the proteasome subunit PSMA1, and the DNA-binding factor THAP12. LDOC1 depletion paradoxically increases global H2Bub1 while reducing its occupancy on chromatin, leading to chromatin compaction and transcriptional repression of metastasis-related genes. Functionally, loss of LDOC1 weakens cell adhesion under TGF-β stimulation and promotes a hybrid E/M state that mirrors the phenotype of STAS-positive tumor cells, highlighting its epigenetic role in controlling tumor cell plasticity and dissemination. Consistent with these mechanistic findings, clinical analyses show that LDOC1 downregulation is associated with elevated H2Bub1 levels in NSCLC specimens, particularly within STAS lesions, and that high H2Bub1 expression correlates with shorter progression-free survival in chemotherapy-treated EGFR^WT^ patients. At the same time, our IHC data reveal substantial heterogeneity, with a subset of LDOC1-high tumors retaining relatively high H2Bub1, underscoring that tumor H2Bub1 levels reflect the integrated output of multiple enzymes and pathways rather than LDOC1 status alone. Together, these observations support a working model in which LDOC1 orchestrates chromatin-associated H2Bub1 dynamics and proteasome-linked histone turnover to regulate tumor plasticity and progression in NSCLC.

**Fig. 7:**
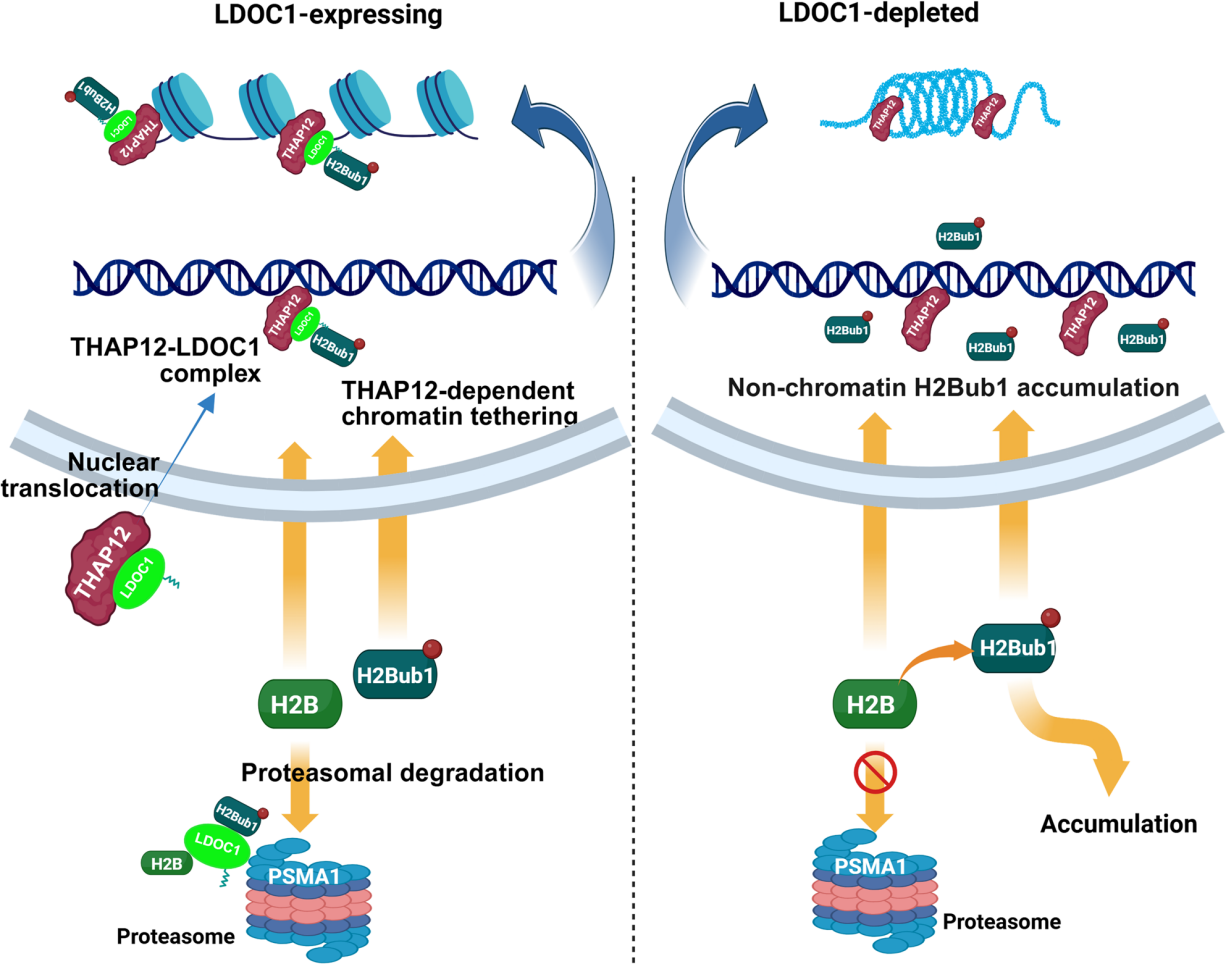
Proposed model of LDOC1-mediated regulation of H2Bub1 and chromatin accessibility via THAP12 and PSMA1. In LDOC1-proficient cells, LDOC1 translocates into the nucleus and is tethered to chromatin through THAP12. The LDOC1–THAP12 complex recruits H2Bub1-marked nucleosomes to accessible regions and couples soluble H2B/H2Bub1 to PSMA1-dependent proteasomal degradation, thereby maintaining focal H2Bub1 enrichment and an open chromatin state. When LDOC1 is depleted, THAP12 can no longer efficiently anchor LDOC1 to chromatin, leading to defective H2Bub1 recruitment, reduced proteasomal turnover of H2B/H2Bub1, accumulation of non-chromatin-bound H2Bub1, global chromatin compaction, and transcriptional reprogramming towards a pro-metastatic state

Mechanistically, our data support a model in which LDOC1 regulates both the abundance and genomic positioning of H2Bub1 through a coordinated interactome (Fig. 7). In LDOC1-expressing NSCLC cells, LDOC1 translocates into the nucleus and binds chromatin by interacting with the DNA-binding factor THAP12. This LDOC1–THAP12 complex recruits H2Bub1-modified nucleosomes to target loci and simultaneously engages the proteasome via PSMA1, promoting controlled degradation of excess soluble H2B/H2Bub1. In this way, LDOC1 restricts the global pool of free H2Bub1 while sustaining its localized enrichment at regulatory regions, thereby preserving chromatin accessibility and epithelial gene expression. Loss of LDOC1 uncouples THAP12 from H2Bub1 and PSMA1, resulting in impaired recruitment of H2Bub1 to chromatin, accumulation of H2B/H2Bub1 in the non-chromatin fraction, chromatin compaction, and activation of EMT and invasion programs. This apparent contradiction is consistent with LDOC1 acting at the interface of ubiquitination and proteasomal turnover: in the absence of LDOC1, H2Bub1 may accumulate in non-chromatin pools while being inefficiently retained at regulatory elements. The highly acidic C-terminal region of LDOC1 (Additional file 1) likely supports transient electrostatic interactions with basic histones, allowing dynamic and context-dependent modulation of H2Bub1 occupancy rather than a static scaffold-like role.

Our findings also begin to clarify the mechanistic link between LDOC1 and THAP12. Because LDOC1 lacks a recognizable DNA-binding domain and canonical NLS, we propose that THAP12, a THAP-family transcription factor with intrinsic DNA-binding and nuclear localization properties, tethers LDOC1 to chromatin. In support of this hierarchy, THAP12 overexpression increases LDOC1 abundance in nuclear histone fractions (Fig. 3j) and drives strong nuclear colocalization (Fig. 3k), and THAP12 overexpression almost abolishes invasion in LDOC1-proficient cells but only partially suppresses the hyper-invasive phenotype of LDOC1-deficient cells (Additional file 7). These observations indicate that LDOC1 is required for the full anti-invasive activity of THAP12 and are consistent with a cooperative LDOC1–THAP12 module that recruits and positions H2Bub1 at chromatin. At the same time, the inability of THAP12 alone to fully rescue LDOC1 loss, together with predicted LDOC1 interactions with additional chromatin regulators such as SMARCD1, suggests that LDOC1 functions within a broader regulatory network rather than a strictly linear pathway.

At the cellular level, LDOC1 emerges as an important regulator of E/M plasticity and adhesion dynamics. LDOC1 depletion induces a hybrid E/M state with co-expression of E-cadherin and mesenchymal markers (Fig. 6a − e), increased motility under basal conditions (Fig. 5c − h), and reduced adhesion under TGF-β stimulation (Fig. 6f). Efficient migration requires a finely tuned cycle of adhesion and release mediated by focal adhesions and cytoskeletal remodeling [30, 31]; LDOC1 loss appears to disturb this balance, weakening adhesion to the point that traction forces become suboptimal and TGF-β-driven migration is paradoxically impaired. Integration of our omics and functional data indicates that LDOC1-dependent control of H2Bub1 and chromatin accessibility directly influences transcriptional programs governing cytoskeletal organization, adhesion, and EMT (Fig. 4c − h), thereby shaping local invasion potential and the emergence of hybrid E/M phenotypes reminiscent of STAS lesions.

Clinically, our IHC analyses demonstrate an inverse association between LDOC1 and H2Bub1 in NSCLC (Fig. 1g, Table 2), particularly within STAS foci (Fig. 6h), and link high H2Bub1 expression to shorter progression-free survival in chemotherapy-treated EGFR^WT^ patients (Fig. 6i). The stronger association of outcome with H2Bub1 than with LDOC1 alone likely reflects that H2Bub1 is a more direct functional readout of chromatin state, whereas LDOC1 is an upstream regulator embedded in a complex interactome. The substantial heterogeneity we observe—LDOC1-high tumors that still retain relatively high H2Bub1—underscores that tumor H2Bub1 levels integrate signals from multiple E3 ligases, deubiquitinases, and chromatin complexes in addition to LDOC1. These considerations support the use of H2Bub1 as a sensitive prognostic biomarker while positioning LDOC1 as a mechanistic node that may help explain why certain tumors acquire highly plastic, STAS-like behavior.

This study has several limitations. We did not perform new in vivo metastasis assays or epistasis experiments using engineered H2B constructs, which would more directly test causality between LDOC1-dependent H2Bub1 remodeling and dissemination. In keeping with the 3R principles and given our prior xenograft work showing that LDOC1 knockdown enhances NSCLC tumorigenicity [21], we chose to prioritize detailed in vitro mechanistic analyses combined with human tumor correlations rather than initiate large additional animal cohorts with uncertain interpretability. Moreover, the precise structural basis of LDOC1’s interactions with chromatin remodelers and the proteasome, as well as the dynamics of LDOC1 regulation in KRAS-mutant versus KRAS^WT^ tumors, remain to be defined. Future work using structural approaches and CRISPR-based epigenome editing to locally manipulate H2Bub1 in vivo will be essential to further refine and causally test the LDOC1–H2Bub1–chromatin axis in NSCLC progression.

## Conclusions

This study identifies LDOC1 as a chromatin-associated tumor suppressor in EGFR^WT^ NSCLC that coordinates H2Bub1 homeostasis, chromatin accessibility, and proteasome-linked histone turnover via an interactome including H2B/H2Bub1, PSMA1, and the DNA-binding factor THAP12. By controlling both the abundance and chromatin recruitment of H2Bub1, LDOC1 maintains transcriptional programs governing cytoskeletal organization, adhesion, and E–M plasticity, thereby limiting the emergence of hybrid E/M states and STAS-like invasive phenotypes. Clinically, LDOC1 downregulation is associated with KRAS mutations and inversely related to H2Bub1 levels in NSCLC, while high H2Bub1 expression predicts shorter progression-free survival in chemotherapy-treated EGFR^WT^ patients. Together, these findings support LDOC1 as a mechanistic hub that restrains tumor plasticity and dissemination, and highlight H2Bub1 as a clinically relevant prognostic marker with potential utility for risk stratification and treatment decision-making in NSCLC.

## Supplementary Information

Supplementary Material 1: Sequences and structural features of LDOC1. (A) Primary amino acid sequence of human LDOC1 (UniProt Q9BUV0), highlighting the leucine zipper domain (residues 11–43, yellow), proline-rich region (residues 61–70, blue), and a highly acidic C-terminal region (residues 115–145, purple). Leucine residues in the zipper motif are indicated in red. (B) Ribbon diagram of AlphaFold-predicted LDOC1 structure (AF-Q9BUV0-F1). (C) Electrostatic surface potential map of LDOC1 rendered in PyMOL using APBS. Red indicates negatively charged and blue indicates positively charged regions. The C-terminal region forms a prominent electronegative surface.

Supplementary Material 2: LDOC1 KD leads to large-scale alterations in gene expression. Scale bars represent the numbers of differentially expressed genes (DEGs) with FC > 2 between NSCLC A549 and PC9 cells with and without LDOC1 depletion.

Supplementary Material 3: Primer sequences used for qRT-PCR.

Supplementary Material 4: Validation of H2Bub1 ChIP–seq profiles and global loss of H2Bub1 occupancy using an independent LDOC1 shRNA in A549 cells. (a) Genome browser tracks showing H2Bub1 ChIP–seq signal across all chromosomes in A549-shCtrl-2 and A549-shLDOC1-2 cells, aligned with RefSeq gene annotations. (b,c) Genomic distribution of H2Bub1 peaks in shCtrl-2 (b; 30,734 peaks) and shLDOC1-2 (c; 4,277 peaks) across promoters, exons, UTRs, introns, and intergenic regions. (d,e) Correlation of H2Bub1 peak intensities between independent experiments for shCtrl (d, shCtrl-1 vs shCtrl-2) and shLDOC1 (e, shLDOC1-1 vs shLDOC1-2) based on log₂-transformed normalized signals (Pearson r = 0.688, n = 20,570 and r = 0.375, n = 3,957 shared peaks, respectively). Each dot represents one peak; red dashed lines indicate y = x. Note the pronounced reduction in both the number and intensity of H2Bub1 peaks upon LDOC1 knockdown, consistent with a global loss of chromatin-bound H2Bub1.

Supplementary Material 5: Validation of LDOC1-dependent chromatin accessibility changes using an independent shRNA in A549 cells. (a) Heatmaps of ATAC-seq signal intensity (±3 kb around peak summits) for unchanged peaks, shLDOC1-2–repressed peaks, and shLDOC1-2–induced peaks in A549-shCtrl-2 versus A549-shLDOC1-2 cells. (b) Genomic distribution of ATAC-seq peaks in each category (unchanged, shLDOC1-2–repressed, shLDOC1-2–induced), annotated according to gene-associated features.

Supplementary Material 6: Ectopic THAP12–GFP expression in A549-shCtrl and A549-shLDOC1 cell pools was verified by fluorescence microscopy (a) and qPCR analysis (b, n = 3).

Supplementary Material 7: Overexpression of THAP12 attenuates invasion of A549 sublines. (a) Representative images of Matrigel-coated transwell invasion assays for A549-shCtrl, shCtrl-THAP12, shLDOC1 and shLDOC1-THAP12 cells after 24 h; invading cells on the lower membrane were fixed and stained with crystal violet (n = 3; two fields per condition shown). (b) Quantification of invading cells (mean ± SD). **p < 0.01, ***p < 0.001.

Supplementary Material 8: Altered transcriptome caused by LDOC1 knockdown in A549 cells. MetaCore pathway enrichment analysis of upregulated (a) and downregulated (b) pathways in A549-shLDOC1 cells based on transcriptomic profiling. Metastasis-related pathways are marked with red stars. (c, d) Heatmaps showing significantly upregulated (c) and downregulated (d) metastasis-related genes in A549-shLDOC1 cells. Fold-change (FC) values were calculated from Affymetrix microarray data; genes with FC > 2 or < –2 and p < 0.05 were considered significant.

Supplementary Material 9: Kaplan–Meier (KM) curves for overall survival (OS) in chemotherapy-treated NSCLC patients stratified by H2Bub1 expression.

Supplementary Material 10.

Supplementary Material 11: Supplementary methods.

## Data Availability

The ChIP-seq and ATAC-seq datasets generated and analyzed during the current study have been deposited in the NCBI Sequence Read Archive (SRA) under BioProject accession number PRJNA1287273 (link), with BioSample accession numbers SAMN49815984. The microarray data are available in the NCBI Gene Expression Omnibus (GEO) under accession numbers GSE235829 (A549-shLDOC1) and GSE298765 (PC9-shLDOC1 and shCtrl). Data will be made publicly accessible upon publication or reasonable request.

## Abbreviation

NSCLC: Non-small cell lung cancer
LDOC1: Leucine Zipper Downregulated in Cancer 1
H2Bub1: H2B monoubiquitination
H3K4me3: Histone H3 trimethylated at lysine 4
ChIP-seq: Chromatin immunoprecipitation followed by sequencing
ATAC-seq: Assay for Transposase-Accessible Chromatin using sequencing
PLA: Proximity ligation assays
CoIP: Co-immunoprecipitation
IHC: Immunohistochemistry
PSMA1: Proteasome Subunit Alpha Type-1
THAP12: *THAP Domain Containing 12*
TGF-β: Transforming growth factor beta
STAS: Spread through air spaces
EGFR: Epidermal growth factor receptor
MNase: Micrococcal nuclease
E/M: Epithelial/Mesenchymal
PFS: Progression-free survival
OS: Overall survival
