# Supplementary material for: LDOC1 connects histone H2B monoubiquitination to tumor cell plasticity in non-small cell lung cancer
Source: Cell Commun Signal. 2026 Jan 3;24:64. doi: 10.1186/s12964-025-02607-z (PMC12853606; doi:10.1186/s12964-025-02607-z)
Supplement: Supplementary file 1 — Supplementary Material 1: Sequences and structural features of LDOC1. (A) Primary amino acid sequence of human LDOC1 (UniProt Q9BUV0), highlighting the leucine zipper domain (residues 11–43, yellow), proline-rich region (residues 61–70, blue), and a highly acidic C-terminal region (residues 115–145, purple). Leucine residues in the zipper motif are indicated in red. (B) Ribbon diagram of AlphaFold-predicted LDOC1 structure (AF-Q9BUV0-F1). (C) Electrostatic surface potential map of LDOC1 rendered in PyMOL using APBS. Red indicates negatively charged and blue indicates positively charged regions. The C-terminal region forms a prominent electronegative surface. [file 12964_2025_2607_MOESM1_ESM.pdf]

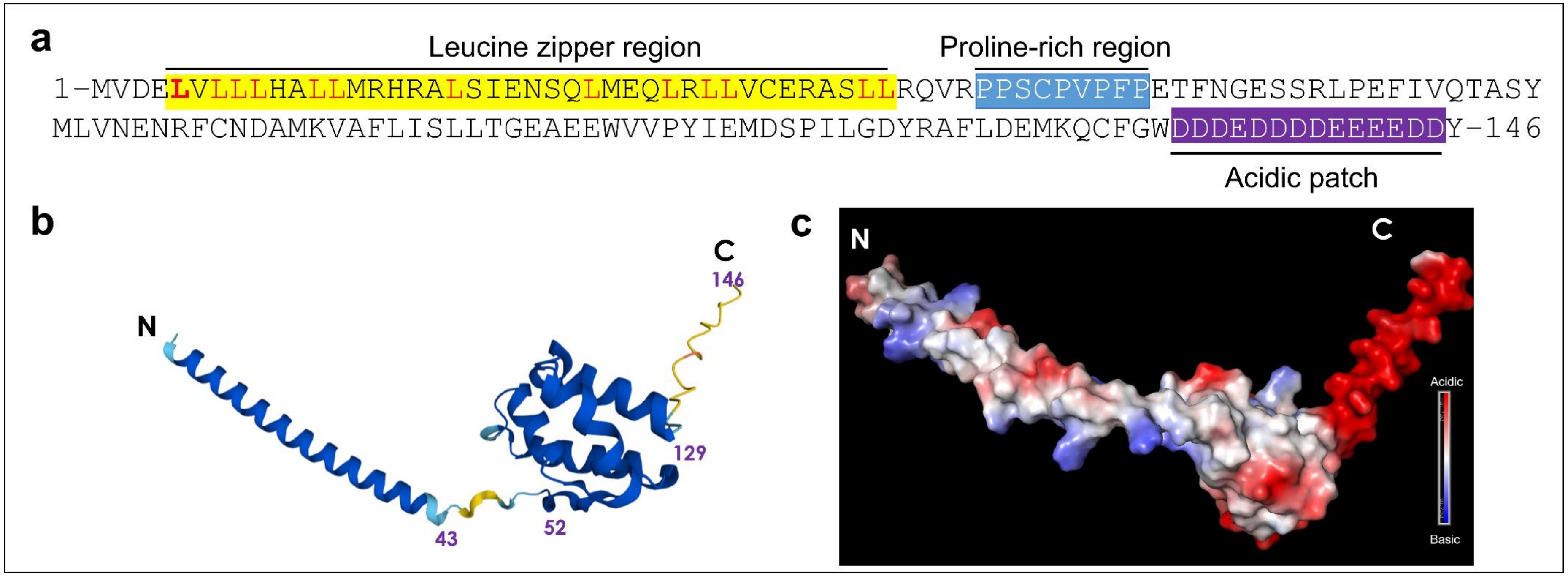
a
Leucine zipper region
Proline-rich region
1-MVDELVLLLHALLMRHRALSIENSQLMEQLRLLVCERASLLRQVRPPSCPVPFPETFNGESSRLPEFIVQTASY
MLVNENRFCNDAMKVAFLISLLTGEAEEWVVPYIEMDSPILGDYRAFLDEMKQCFGWDDDEDDDDEEEEDDY-146
Acidic patch
b
N
C
146
129
43
52
c
N
C
Acidic
Basic
