# Supplementary figures and images for: LDOC1 connects histone H2B monoubiquitination to tumor cell plasticity in non-small cell lung cancer
Source: Cell Commun Signal. 2026 Jan 3;24:64. doi: 10.1186/s12964-025-02607-z (PMC12853606; doi:10.1186/s12964-025-02607-z)

## Differential Expressed Genes

**A549**  
**shLDOC1 vs. shCtrl**  
**(GSE235829)**

**PC9**  
**shLDOC1 vs. shCtrl**  
**(GSE298765)**

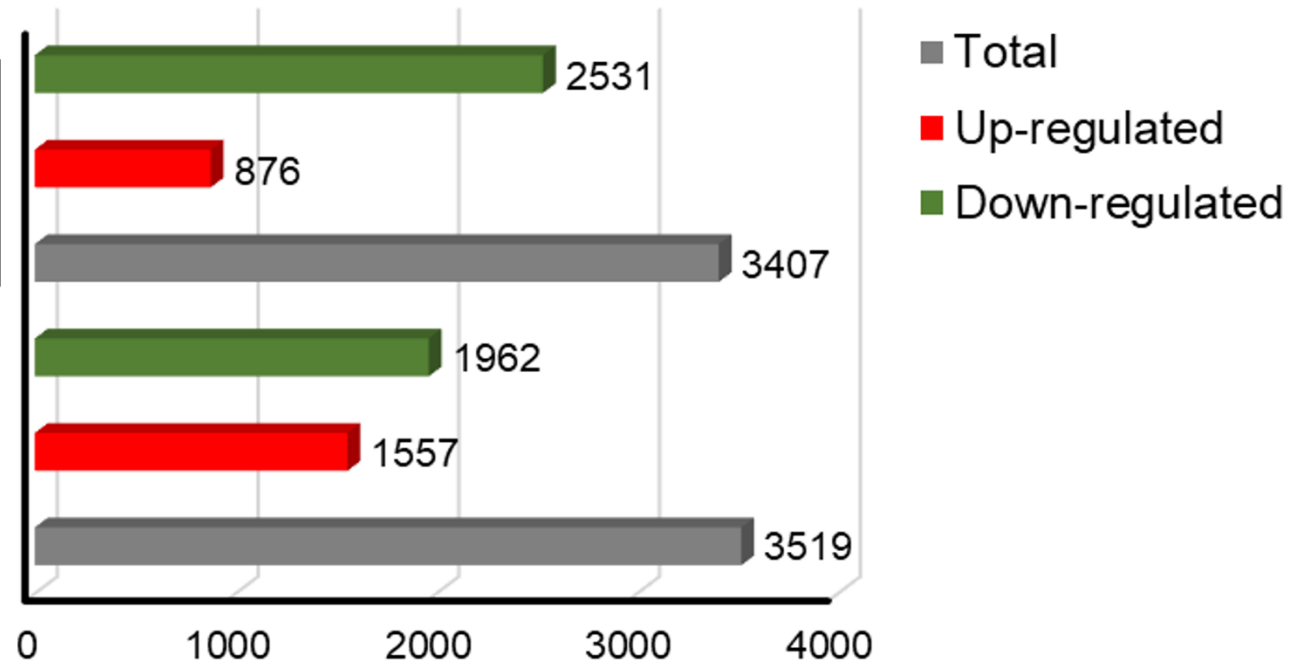

Supplement: Supplementary file 2 — Supplementary Material 2: LDOC1 KD leads to large-scale alterations in gene expression. Scale bars represent the numbers of differentially expressed genes (DEGs) with FC > 2 between NSCLC A549 and PC9 cells with and without LDOC1 depletion. [file 12964_2025_2607_MOESM2_ESM.pdf]

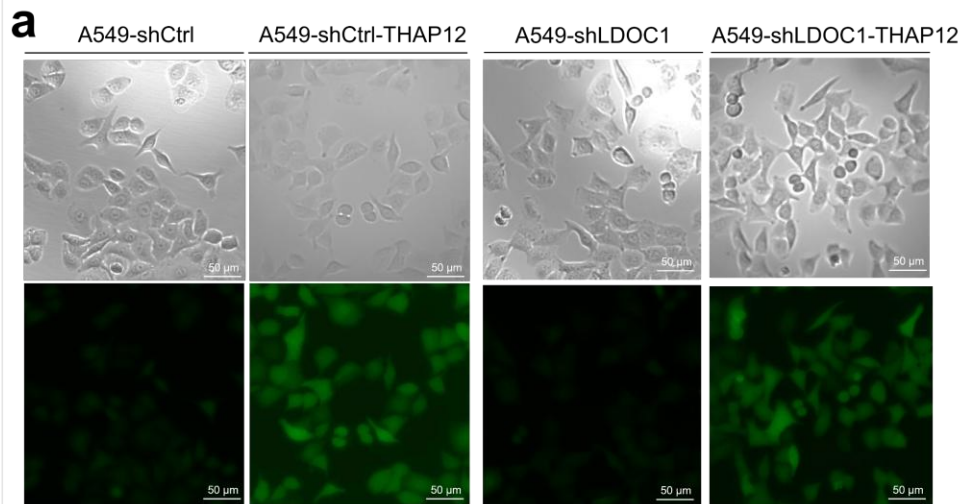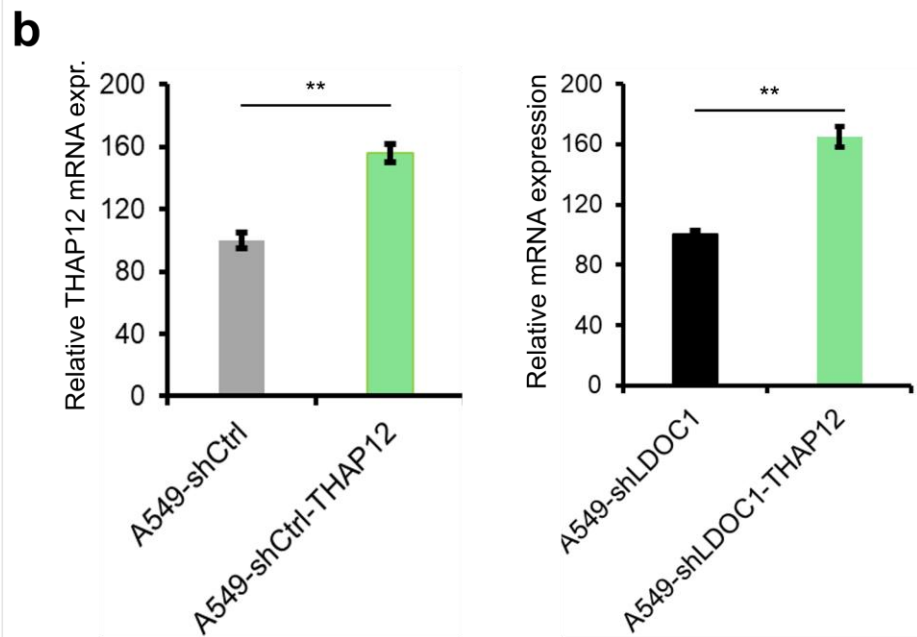

Supplement: Supplementary file 6 — Supplementary Material 6: Ectopic THAP12–GFP expression in A549-shCtrl and A549-shLDOC1 cell pools was verified by fluorescence microscopy (a) and qPCR analysis (b, n = 3). [file 12964_2025_2607_MOESM6_ESM.pdf]

**a**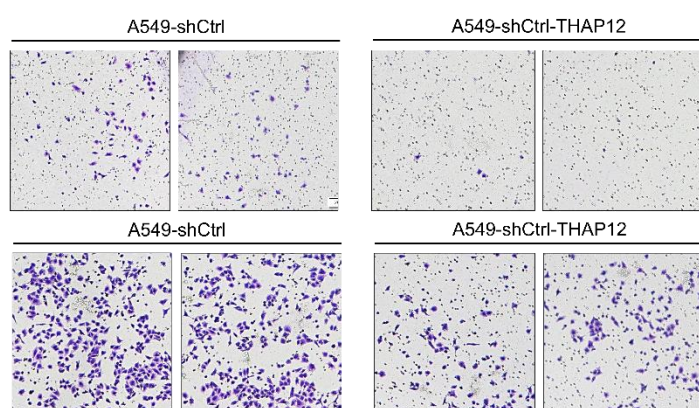**b**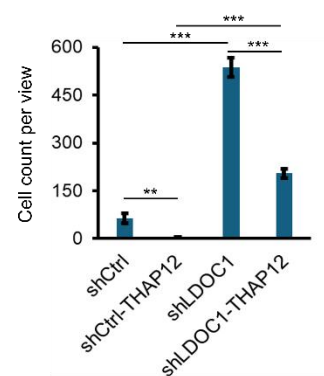

Supplement: Supplementary file 7 — Supplementary Material 7: Overexpression of THAP12 attenuates invasion of A549 sublines. (a) Representative images of Matrigel-coated transwell invasion assays for A549-shCtrl, shCtrl-THAP12, shLDOC1 and shLDOC1-THAP12 cells after 24 h; invading cells on the lower membrane were fixed and stained with crystal violet (n = 3; two fields per condition shown). (b) Quantification of invading cells (mean ± SD). **p < 0.01, ***p < 0.001. [file 12964_2025_2607_MOESM7_ESM.pdf]

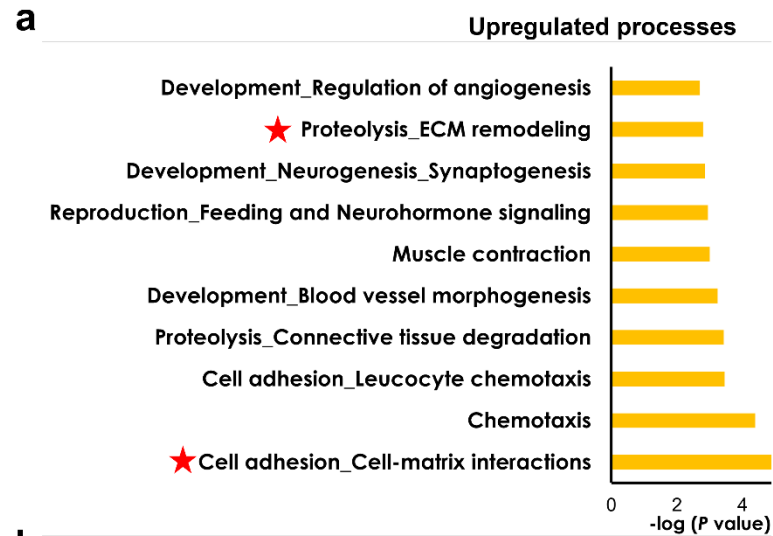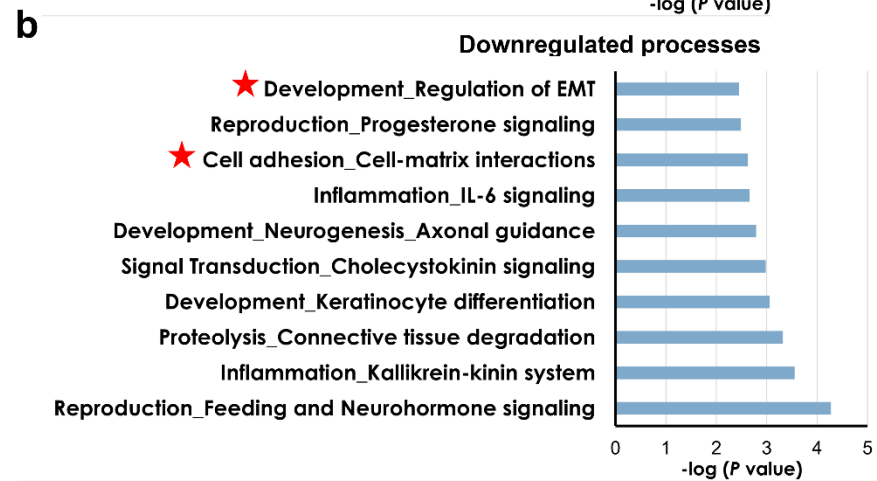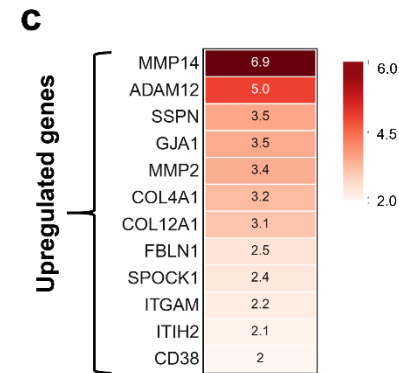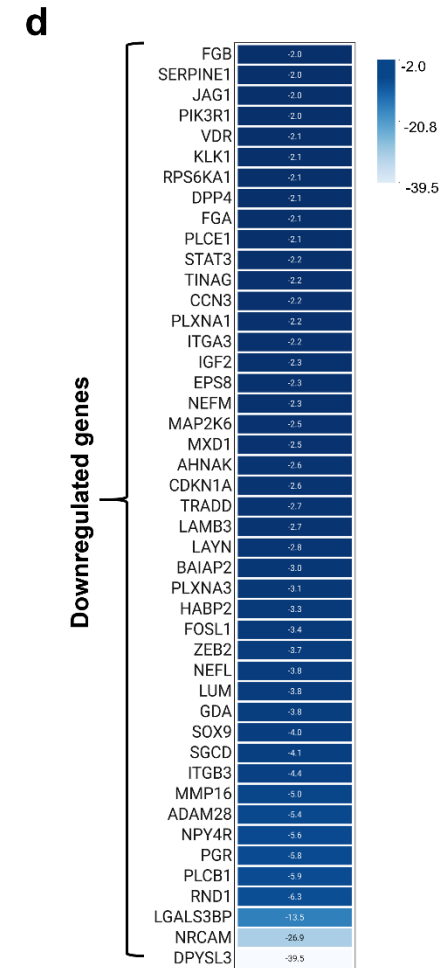

Supplement: Supplementary file 8 — Supplementary Material 8: Altered transcriptome caused by LDOC1 knockdown in A549 cells. MetaCore pathway enrichment analysis of upregulated (a) and downregulated (b) pathways in A549-shLDOC1 cells based on transcriptomic profiling. Metastasis-related pathways are marked with red stars. (c, d) Heatmaps showing significantly upregulated (c) and downregulated (d) metastasis-related genes in A549-shLDOC1 cells. Fold-change (FC) values were calculated from Affymetrix microarray data; genes with FC > 2 or < –2 and p < 0.05 were considered significant. [file 12964_2025_2607_MOESM8_ESM.pdf]

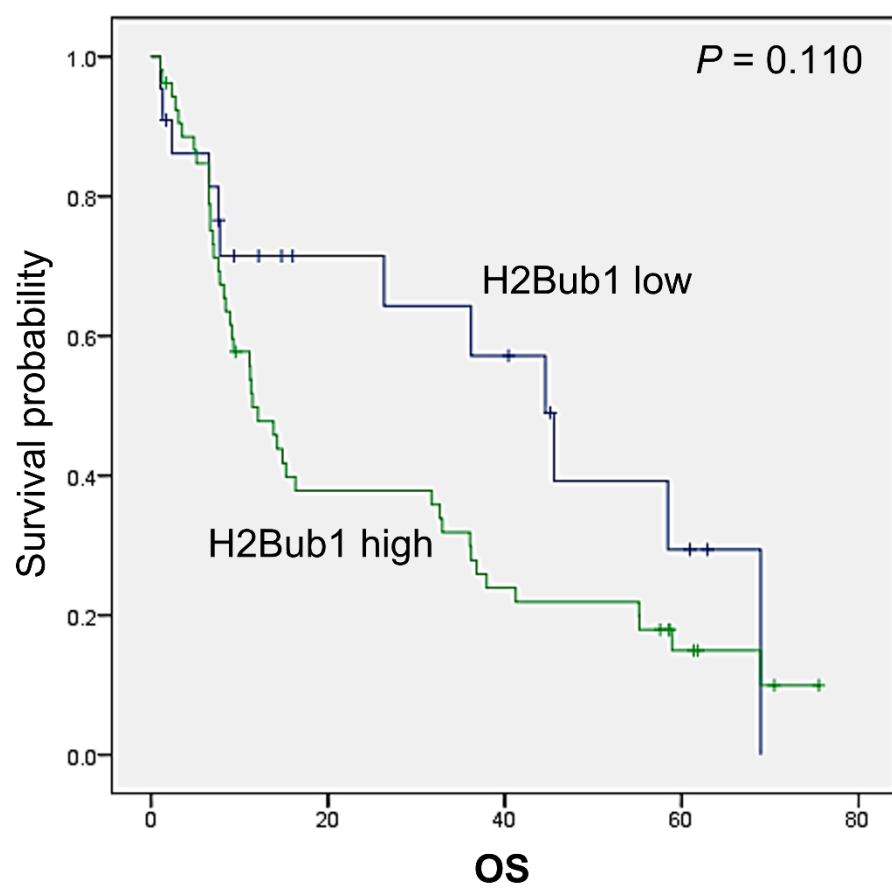

Supplement: Supplementary file 9 — Supplementary Material 9: Kaplan–Meier (KM) curves for overall survival (OS) in chemotherapy-treated NSCLC patients stratified by H2Bub1 expression. [file 12964_2025_2607_MOESM9_ESM.pdf]
