# Supplementary material for: LDOC1 connects histone H2B monoubiquitination to tumor cell plasticity in non-small cell lung cancer
Source: Cell Commun Signal. 2026 Jan 3;24:64. doi: 10.1186/s12964-025-02607-z (PMC12853606; doi:10.1186/s12964-025-02607-z)
Supplement: Supplementary file 3 — Supplementary Material 3: Primer sequences used for qRT-PCR. [file 12964_2025_2607_MOESM3_ESM.pdf]

Additional file 3. Primer sequences used for qRT-PCR.

Cdh1–F	5'-GCCTCCTGAAAAGAGAGTGGAAG-3'
Cdh1–R	5'-TGGCAGTGTCTCTCCAAATCCG-3'
Cdh2–F	5'-CCTCCAGAGTTTACTGCCATGAC-3'
Cdh2–R	5'-GTAGGATCTCCGCCACTGATTC-3'
Vim–F	5'-AGGCAAAGCAGGAGTCCACTGA-3'
Vim–R	5'-ATCTGGCGTTCCAGGGACTCAT-3'
Itga3–F	5'-GCCTGACAACAAGTGTGAGAGC-3'
Itga3–R	5'-GGTGTTCGTCACGTTGATGCTC-3'
Flnb–F	5'-CCTTCAAGGTGGCTGTCACTGA-3'
Flnb–R	5'-CCCTCAACAGTTATGCCAAGCC-3'
Arhgdib–F	5'-CGTTCAGCACACCTACAGGACT-3'
Arhgdib–R	5'-TTGGGAGCCTCCTCAACTGGAG-3'
Gapdh–F	5'-GTCTCCTCTGACTTCAACAGCG-3'
Gapdh–R	5'-ACCACCCTGTTGCTGTAGCCAA-3'
