# Supplementary material for: LDOC1 connects histone H2B monoubiquitination to tumor cell plasticity in non-small cell lung cancer
Source: Cell Commun Signal. 2026 Jan 3;24:64. doi: 10.1186/s12964-025-02607-z (PMC12853606; doi:10.1186/s12964-025-02607-z)
Supplement: Supplementary file 4 — Supplementary Material 4: Validation of H2Bub1 ChIP–seq profiles and global loss of H2Bub1 occupancy using an independent LDOC1 shRNA in A549 cells. (a) Genome browser tracks showing H2Bub1 ChIP–seq signal across all chromosomes in A549-shCtrl-2 and A549-shLDOC1-2 cells, aligned with RefSeq gene annotations. (b,c) Genomic distribution of H2Bub1 peaks in shCtrl-2 (b; 30,734 peaks) and shLDOC1-2 (c; 4,277 peaks) across promoters, exons, UTRs, introns, and intergenic regions. (d,e) Correlation of H2Bub1 peak intensities between independent experiments for shCtrl (d, shCtrl-1 vs shCtrl-2) and shLDOC1 (e, shLDOC1-1 vs shLDOC1-2) based on log₂-transformed normalized signals (Pearson r = 0.688, n = 20,570 and r = 0.375, n = 3,957 shared peaks, respectively). Each dot represents one peak; red dashed lines indicate y = x. Note the pronounced reduction in both the number and intensity of H2Bub1 peaks upon LDOC1 knockdown, consistent with a global loss of chromatin-bound H2Bub1. [file 12964_2025_2607_MOESM4_ESM.pdf]

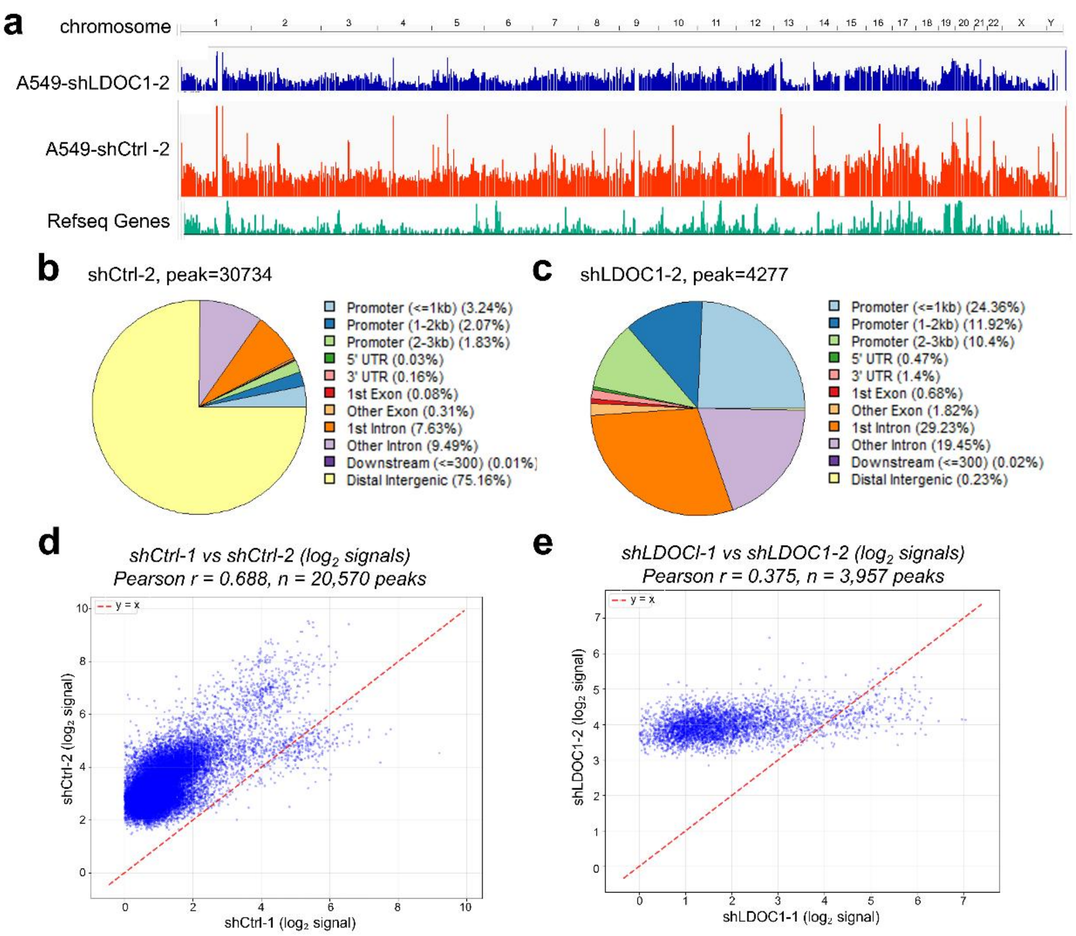
a
chromosome
1 2 3 4 5 6 7 8 9 10 11 12 13 14 15 16 17 18 19 20 21 22 X Y
A549-shLDOC1-2
A549-shCtrl -2
Refseq Genes
b
shCtrl-2, peak=30734
Promoter (<=1kb) (3.24%)
Promoter (1-2kb) (2.07%)
Promoter (2-3kb) (1.83%)
5' UTR (0.03%)
3' UTR (0.16%)
1st Exon (0.08%)
Other Exon (0.31%)
1st Intron (7.63%)
Other Intron (9.49%)
Downstream (<=300) (0.01%)
Distal Intergenic (75.16%)
c
shLDOC1-2, peak=4277
Promoter (<=1kb) (24.36%)
Promoter (1-2kb) (11.92%)
Promoter (2-3kb) (10.4%)
5' UTR (0.47%)
3' UTR (1.4%)
1st Exon (0.68%)
Other Exon (1.82%)
1st Intron (29.23%)
Other Intron (19.45%)
Downstream (<=300) (0.02%)
Distal Intergenic (0.23%)
d
shCtrl-1 vs shCtrl-2 (log2 signals)
Pearson r = 0.688, n = 20,570 peaks
y = x
shCtrl-2 (log2 signal)
shCtrl-1 (log2 signal)
e
shLDOCl-1 vs shLDOC1-2 (log2 signals)
Pearson r = 0.375, n = 3,957 peaks
y = x
shLDOC1-2 (log2 signal)
shLDOC1-1 (log2 signal)
