# Supplementary material for: LDOC1 connects histone H2B monoubiquitination to tumor cell plasticity in non-small cell lung cancer
Source: Cell Commun Signal. 2026 Jan 3;24:64. doi: 10.1186/s12964-025-02607-z (PMC12853606; doi:10.1186/s12964-025-02607-z)
Supplement: Supplementary file 5 — Supplementary Material 5: Validation of LDOC1-dependent chromatin accessibility changes using an independent shRNA in A549 cells. (a) Heatmaps of ATAC-seq signal intensity (±3 kb around peak summits) for unchanged peaks, shLDOC1-2–repressed peaks, and shLDOC1-2–induced peaks in A549-shCtrl-2 versus A549-shLDOC1-2 cells. (b) Genomic distribution of ATAC-seq peaks in each category (unchanged, shLDOC1-2–repressed, shLDOC1-2–induced), annotated according to gene-associated features. [file 12964_2025_2607_MOESM5_ESM.pdf]

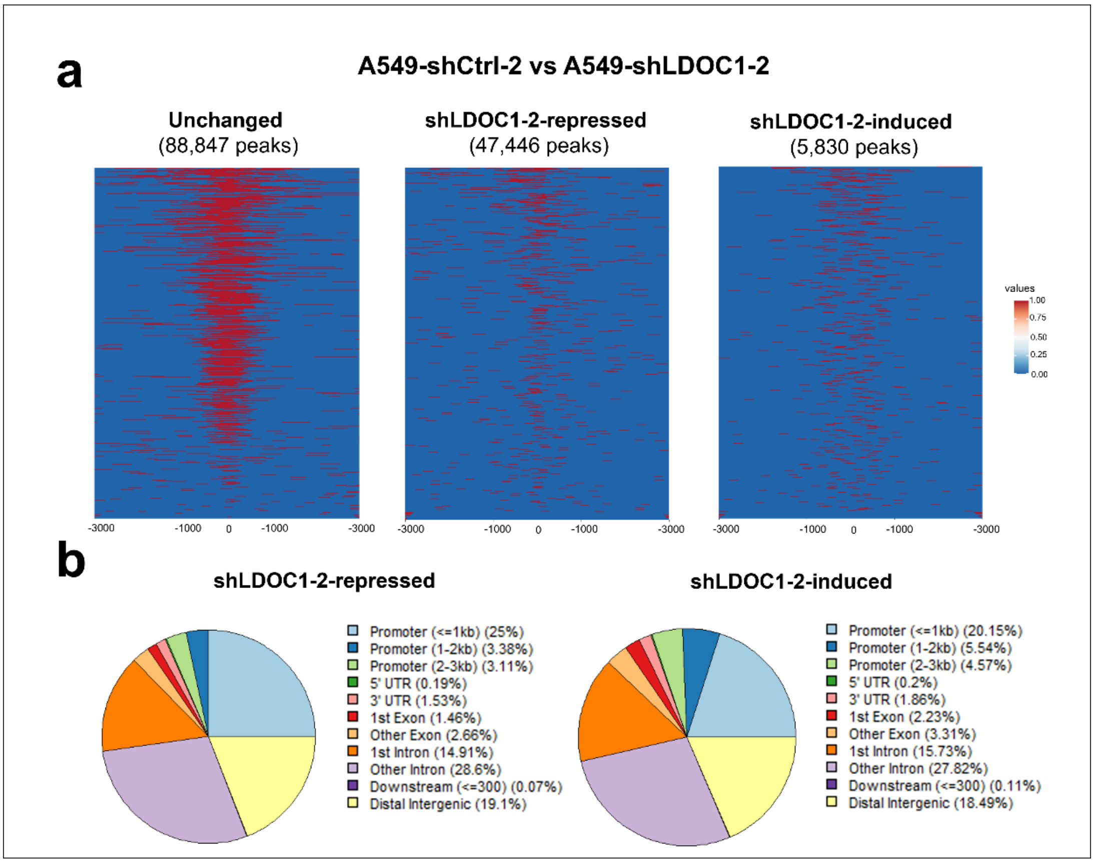
a
A549-shCtrl-2 vs A549-shLDOC1-2
Unchanged
(88,847 peaks)
shLDOC1-2-repressed
(47,446 peaks)
shLDOC1-2-induced
(5,830 peaks)
values
1.00
0.75
0.50
0.25
0.00
-3000
-1000
0
-1000
-3000
b
shLDOC1-2-repressed
Promoter (<=1kb) (25%)
Promoter (1-2kb) (3.38%)
Promoter (2-3kb) (3.11%)
5' UTR (0.19%)
3' UTR (1.53%)
1st Exon (1.46%)
Other Exon (2.66%)
1st Intron (14.91%)
Other Intron (28.6%)
Downstream (<=300) (0.07%)
Distal Intergenic (19.1%)
shLDOC1-2-induced
Promoter (<=1kb) (20.15%)
Promoter (1-2kb) (5.54%)
Promoter (2-3kb) (4.57%)
5' UTR (0.2%)
3' UTR (1.86%)
1st Exon (2.23%)
Other Exon (3.31%)
1st Intron (15.73%)
Other Intron (27.82%)
Downstream (<=300) (0.11%)
Distal Intergenic (18.49%)
