# Supplementary material for: LDOC1 connects histone H2B monoubiquitination to tumor cell plasticity in non-small cell lung cancer
Source: Cell Commun Signal. 2026 Jan 3;24:64. doi: 10.1186/s12964-025-02607-z (PMC12853606; doi:10.1186/s12964-025-02607-z)

Fig. 1e Whole-cell lysates

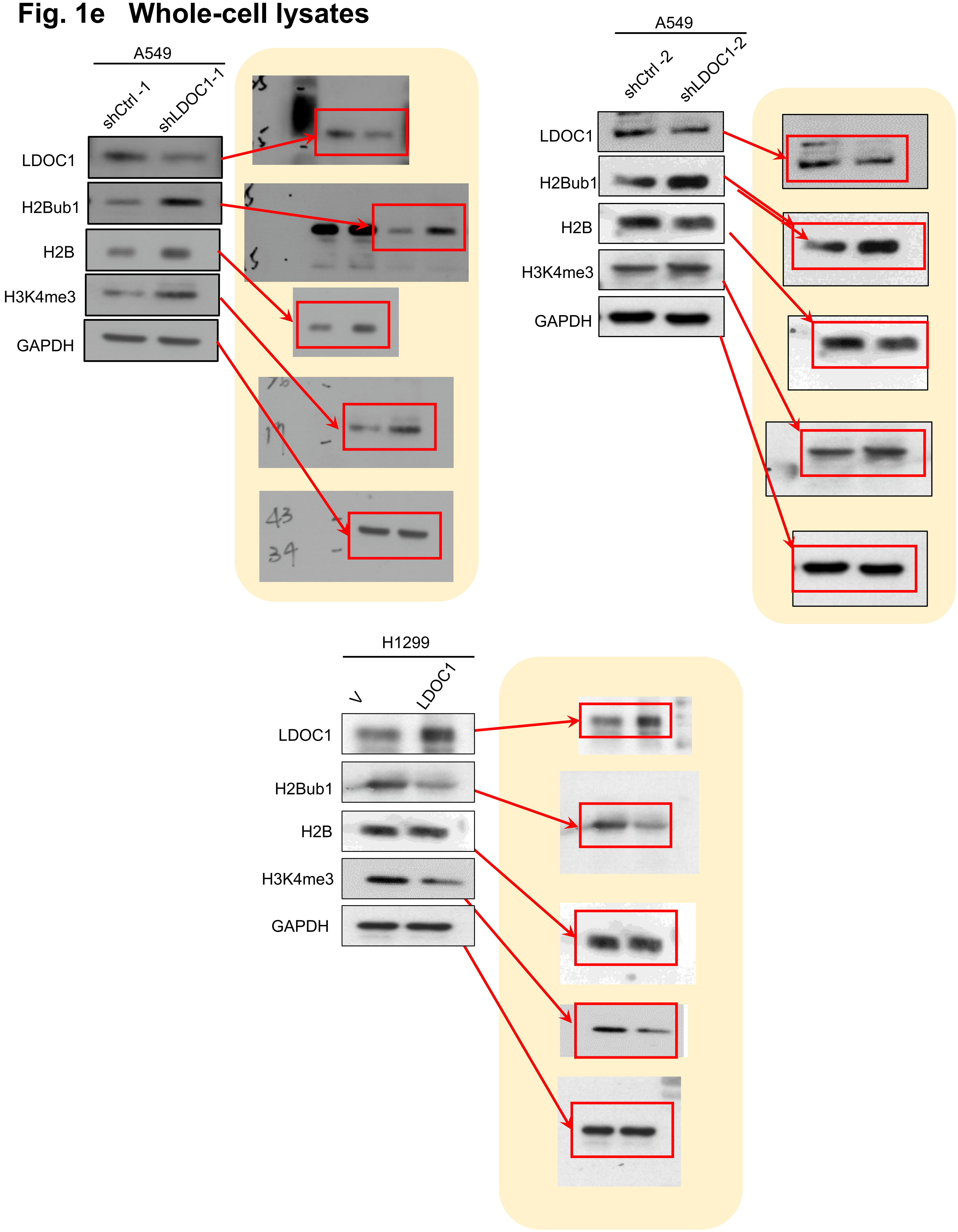

Fig. 1f Nuclear histone extracts

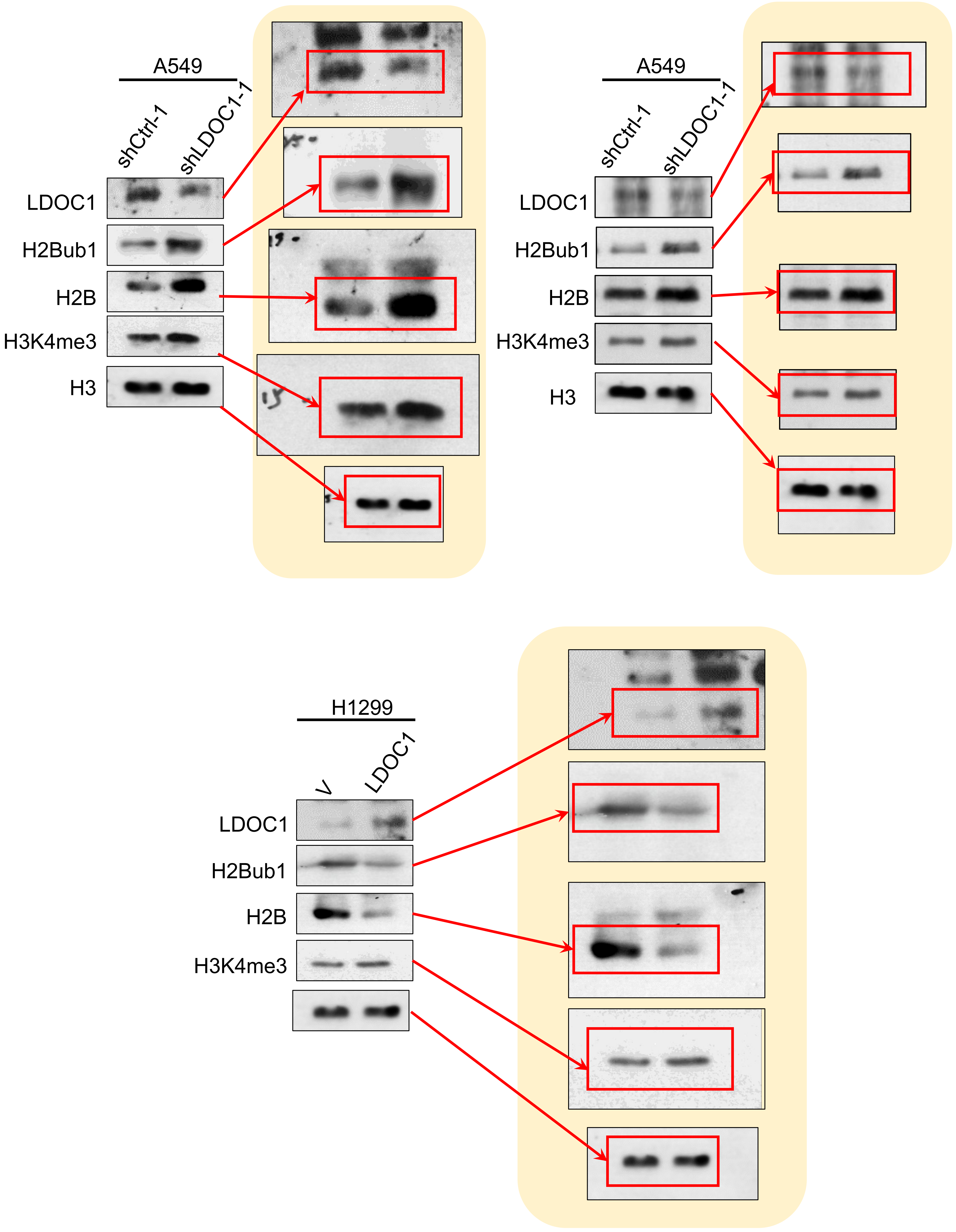

Fig. 1h

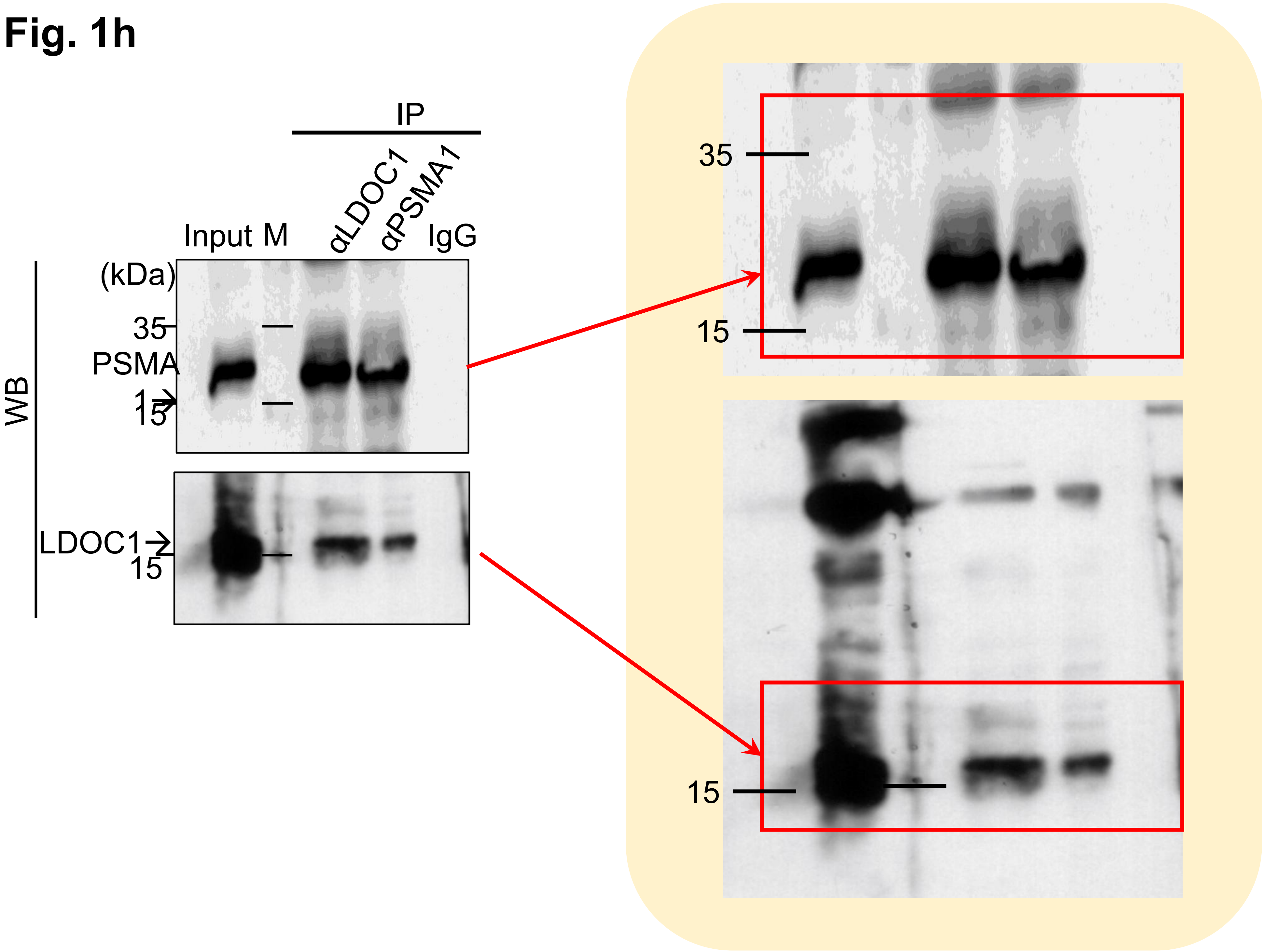

IP
αLDOC1
αPSMA1
Input
M
IgG
(kDa)
35
PSMA1
15
WB
LDOC1
15
35
15
15

Fig. 1j

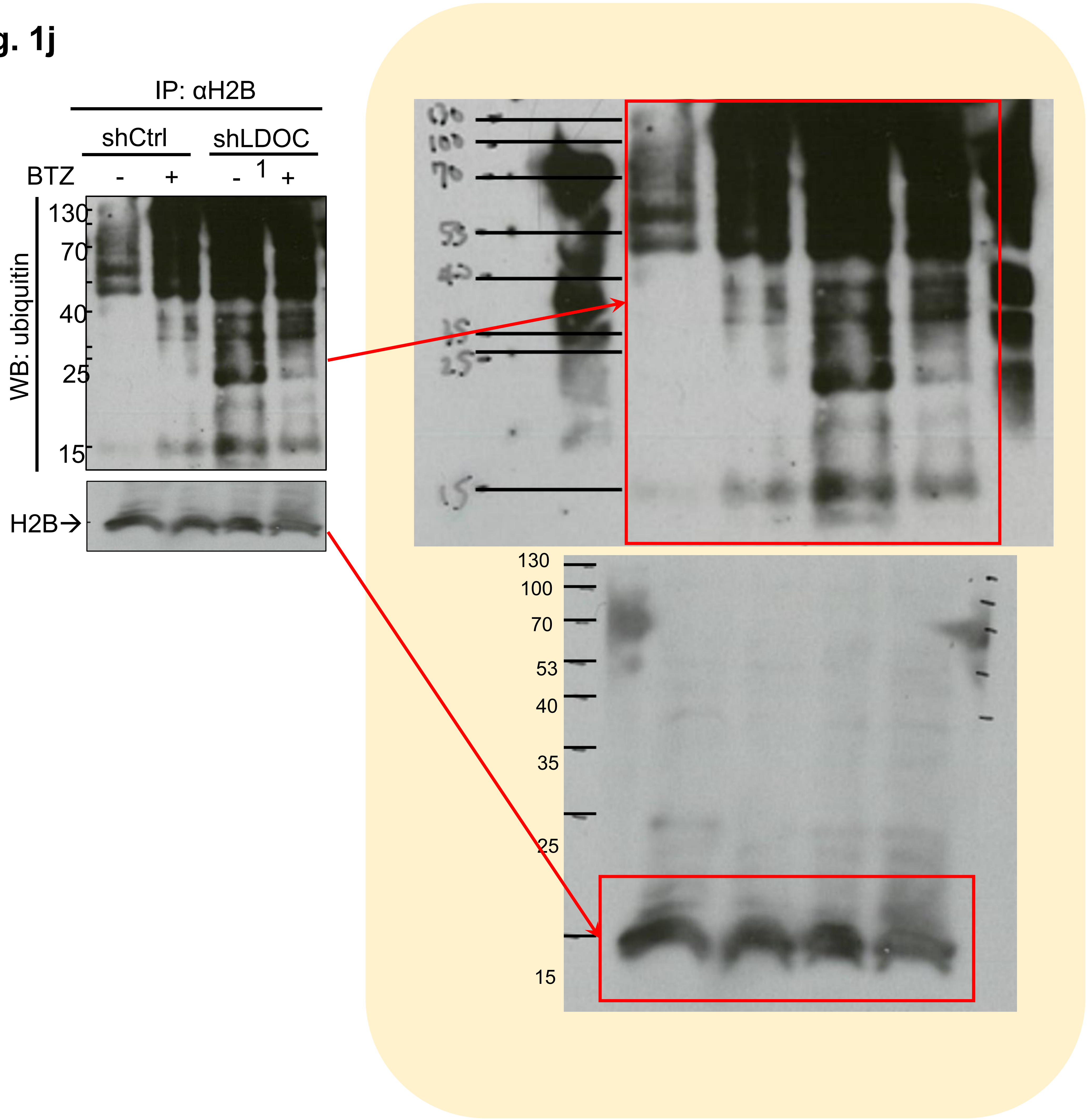

IP: αH2B
shCtrl
shLDOC 1
BTZ - + - +
WB: ubiquitin
130
70
40
25
15
H2B
130
100
70
53
40
35
25
15

Fig. 1k

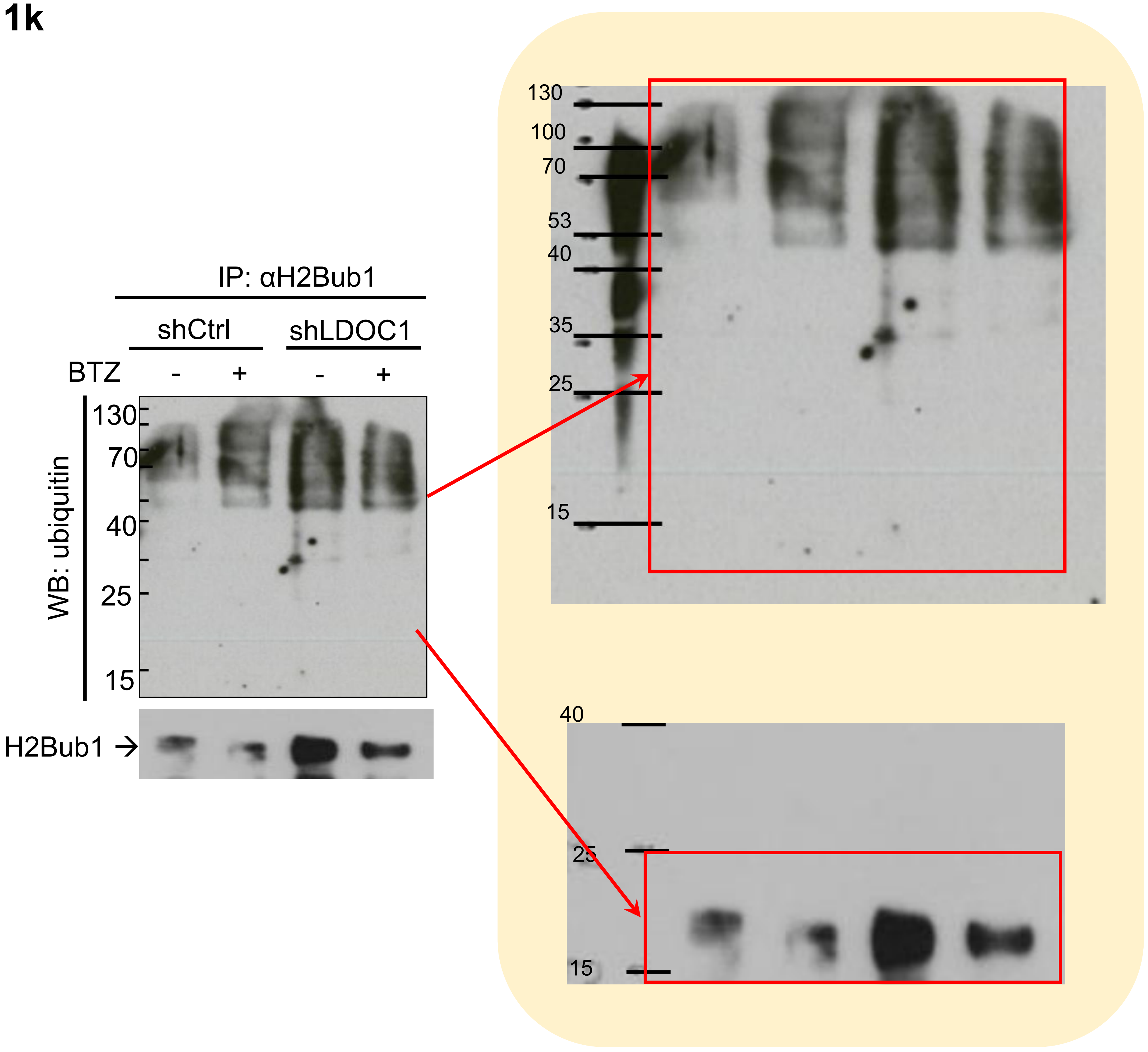

IP: αH2Bub1
shCtrl
shLDOC1
BTZ
-
+
-
+
WB: ubiquitin
130
70
40
25
15
H2Bub1
130
100
70
53
40
35
25
15
40
25
15

Fig. 3f

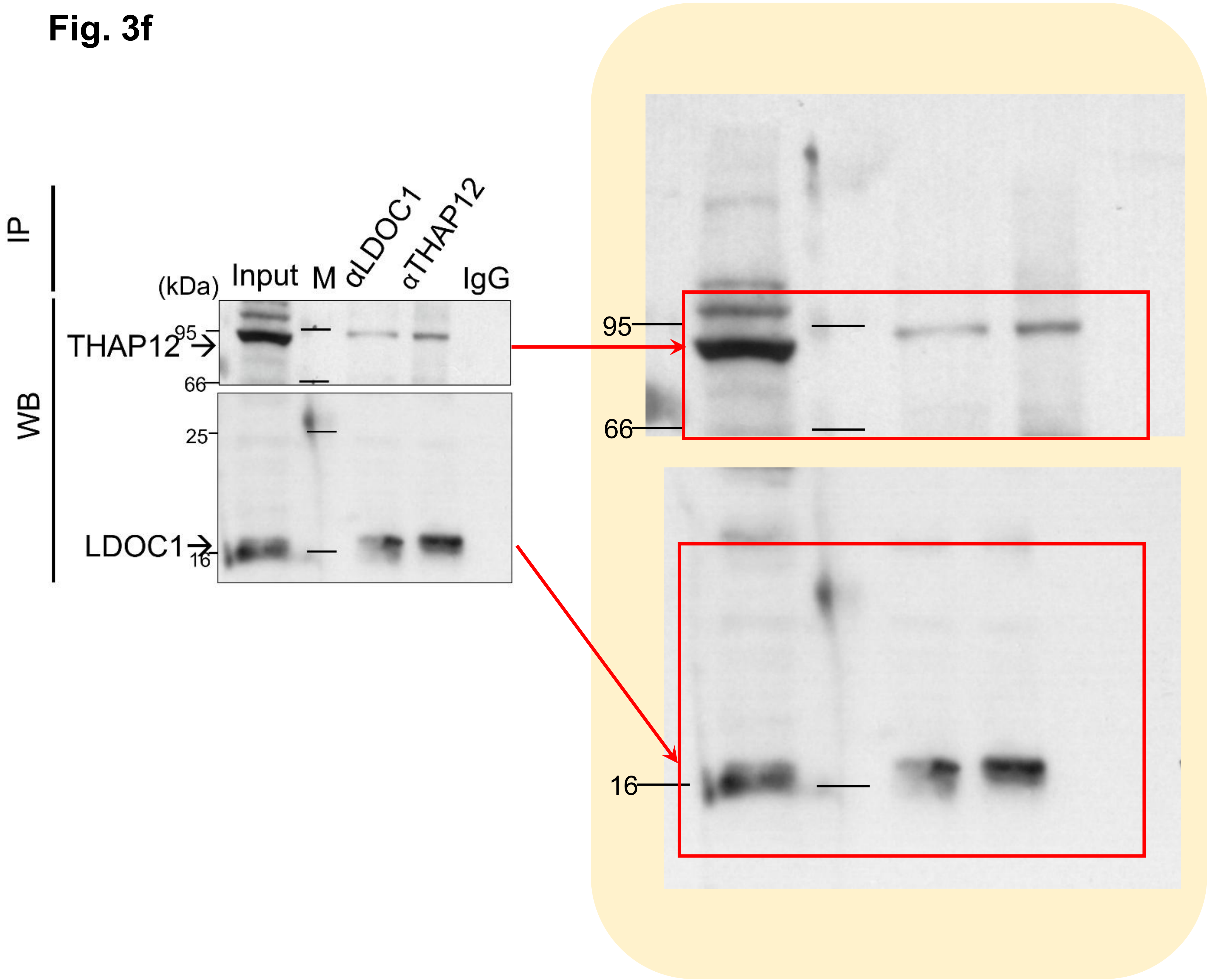
IP
WB
(kDa)
Input
M
αLDOC1
αTHAP12
IgG
THAP12
95
66
25
LDOC1
16
95
66
16

Fig. 3j

Nuclear histone extract

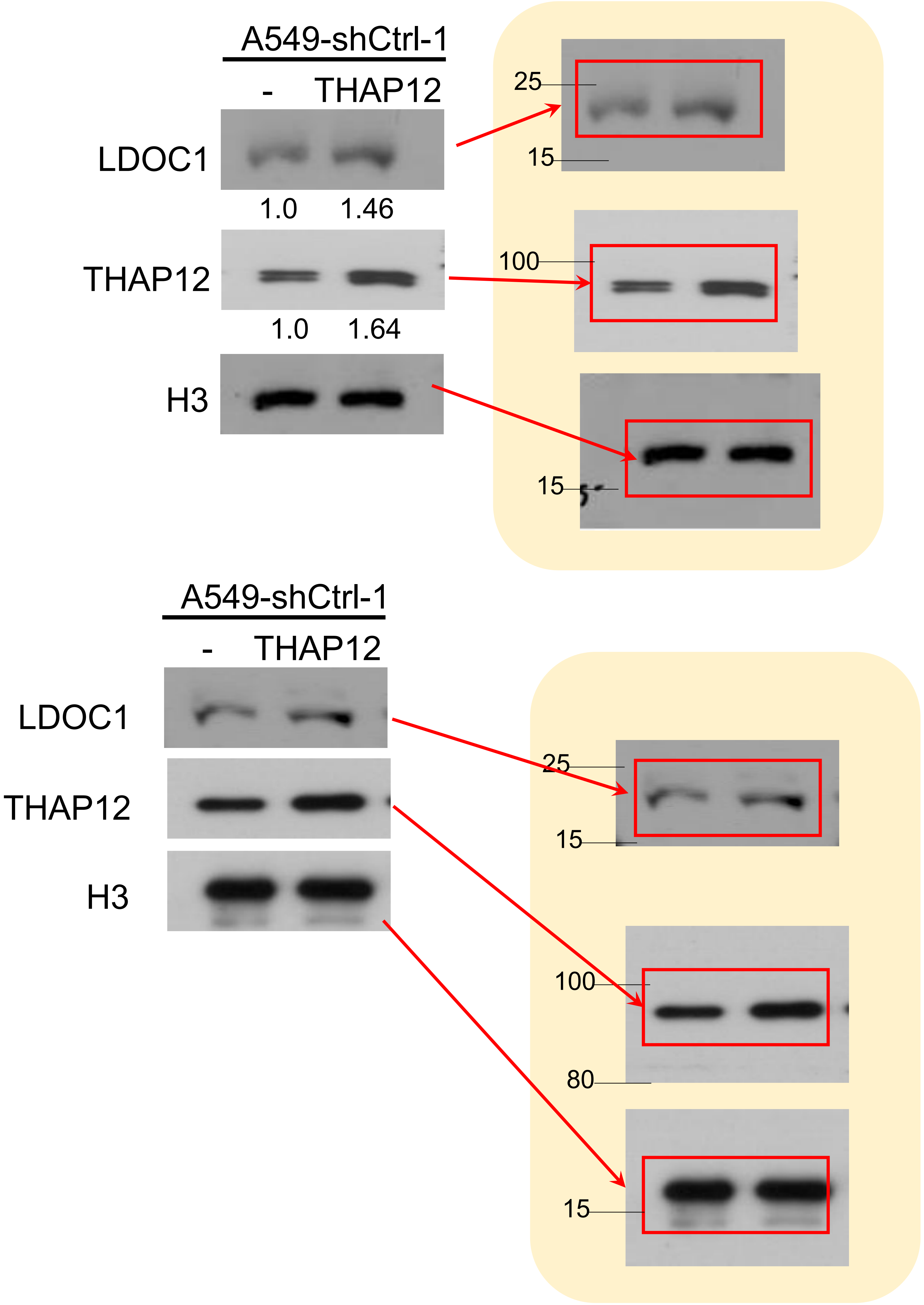

Supplement: Supplementary file 10 — Supplementary Material 10. [file 12964_2025_2607_MOESM10_ESM.pdf]
