# Supplementary material for: LDOC1 connects histone H2B monoubiquitination to tumor cell plasticity in non-small cell lung cancer
Source: Cell Commun Signal. 2026 Jan 3;24:64. doi: 10.1186/s12964-025-02607-z (PMC12853606; doi:10.1186/s12964-025-02607-z)
Supplement: Supplementary file 11 — Supplementary Material 11: Supplementary methods. [file 12964_2025_2607_MOESM11_ESM.docx]

**Supplementary Methods**

**Establishment of stable LDOC1-knockdown cell lines**

Lentiviral vectors (pLAS2w.Ppuro) carrying shRNAs targeting human LDOC1 were used to generate two independent knockdown sublines. The target sequences were A549-shLDOC1-1, 5′-GCCTTCCTCGATGAGATGAAA-3′ (TRCN0000118178), and A549-shLDOC1-2, 5′-GCTCGTGAACGAGAACCGATT-3′ (TRCN0000118179). In parallel, control cell lines (A549-shCtrl-1 and A549-shCtrl-2) were established using lentiviruses encoding a scrambled shRNA sequence. The corresponding lentiviral particles were generated by the National RNAi Core Facility at Academia Sinica (Taipei, Taiwan) and used to transduce A549 cells at a multiplicity of infection (MOI) of approximately 2 in the presence of 8 µg/mL polybrene. After 24 h, the medium was replaced, and transduced cells were selected with puromycin (5 µg/mL) for approximately one weeks until all non-infected cells had died. Following antibiotic selection, two independent stable clones were isolated for each construct and expanded, yielding A549-shLDOC1-1, A549-shLDOC1-2, A549-shCtrl-1, and A549-shCtrl-2. Knockdown efficiency was validated by quantitative RT-PCR and immunoblotting using anti-LDOC1 antibody (customized), with GAPDH or β-actin as loading controls. Only clones showing ≥ 40 % reduction in LDOC1 expression at both mRNA and protein levels were used for subsequent experiments. All cell lines were confirmed to be free of mycoplasma contamination.

**Single- and double-immunofluorescence staining**Cells on glass coverslips were fixed with 4% paraformaldehyde for 15 min at room temperature, permeabilized in PBS with 0.1% Triton X-100 for 10 min, and blocked in 3% BSA/PBS for 1 h. Primary antibodies were fluorescently labeled using the FlexAble Antibody Labeling Kit (according to the manufacturer’s instructions) and incubated overnight at 4 °C: anti-LDOC1 (1:500; red), anti-H2B (0.1 µg/mL; green), anti-H2Bub1 (1:2500; green), and—for single-label staining—anti-E-cadherin (1:2000). After PBS washes, nuclei were counterstained with DAPI, and coverslips were mounted in EverBrite™ mounting medium. Images were acquired on a Leica STELLARIS 8 confocal microscope using identical laser and detector settings across samples.

**Repeated H2Bub1 ChIP-seq Library Preparation and Sequencing**

The repeated H2Bub1 ChIP-seq library preparation and sequencing for A549-shCtrl-2 and A549-shLDOC1-2 cells were performed by the High Throughput Genomics Core at the Biodiversity Research Center, Academia Sinica, using an Illumina NextSeq 2000 platform with a P3 flow cell (single-end 50-bp reads; four multiplexed libraries per lane) according to the core facility’s standard protocols.

**Generation of THAP12–GFP–expressing A549 cell pools**Lentiviral particles encoding human THAP12 (PRKRIR; NM_004705) fused to monomeric GFP in the pLenti-C-mGFP-P2A-Puro backbone (OriGene, RC210314L4V) were used to generate THAP12–GFP–expressing pools from A549-shCtrl and A549-shLDOC1 cells. Cells were seeded at ~40–50% confluence and infected with the THAP12–GFP lentivirus in complete growth medium containing polybrene. After 24 h, the virus-containing medium was replaced with fresh medium, and cells were allowed to recover and expand without antibiotic selection because both A549-shCtrl and A549-shLDOC1 cells already harbor a puromycin resistance cassette. Infection and expression efficiency were monitored by fluorescence microscopy, and only cultures in which >70% of cells exhibited clear nuclear GFP fluorescence were used as THAP12–GFP–expressing pools for subsequent experiments.

**Transwell migration/invasion assay**
Transwell inserts (8-µm pores, 24-well) were equilibrated before use. Cells were serum-starved for 16 h, resuspended in serum-free medium, and seeded at 8 × 10⁴ cells in 150 µL into the upper chamber; 700 µL medium containing 10% FBS was added to the lower chamber as chemoattractant. After 24 h at 37 °C/5% CO₂, cells remaining on the upper surface were removed. Membranes were fixed in methanol (10 min), stained with 0.1% crystal violet (15 min), rinsed, air-dried, and migrated cells on the underside were imaged in five random fields at 200× and counted. Data are presented as mean ± SEM from ≥3 independent experiments and normalized to the control group. For invasion assays, the same procedure was used with Matrigel-coated inserts.
